# Volcanically driven lacustrine ecosystem changes during the Carnian Pluvial Episode (Late Triassic)

**DOI:** 10.1073/pnas.2109895118

**Published:** 2021-09-27

**Authors:** Jing Lu, Peixin Zhang, Jacopo Dal Corso, Minfang Yang, Paul B. Wignall, Sarah E. Greene, Longyi Shao, Dan Lyu, Jason Hilton

**Affiliations:** ^a^State Key Laboratory of Coal Resources and Safe Mining, College of Geoscience and Surveying Engineering, China University of Mining and Technology, Beijing 100083, PR China;; ^b^State Key Laboratory of Biogeology and Environmental Geology, China University of Geosciences, Wuhan 430074, PR China;; ^c^Research Institute of Petroleum Exploration and Development, PetroChina, Beijing 100083, PR China;; ^d^School of Earth and Environment, University of Leeds, Leeds LS2 9JT, United Kingdom;; ^e^School of Geography, Earth, and Environmental Sciences, University of Birmingham, Birmingham B15 2TT, United Kingdom;; ^f^Key Laboratory of Oil and Gas Reservoir, China National Petroleum Corporation, Beijing 100083, PR China

**Keywords:** large igneous province, volcanism, Triassic, Carnian Pluvial Episode, climate change

## Abstract

The Late Triassic Carnian Pluvial Episode (CPE) saw a dramatic increase in global humidity and temperature that has been linked to the large-scale volcanism of the Wrangellia large igneous province. The climatic changes coincide with a major biological turnover on land that included the ascent of the dinosaurs and the origin of modern conifers. However, linking the disparate cause and effects of the CPE has yet to be achieved because of the lack of a detailed terrestrial record of these events. Here, we present a multidisciplinary record of volcanism and environmental change from an expanded Carnian lake succession of the Jiyuan Basin, North China. New U–Pb zircon dating, high-resolution chemostratigraphy, and palynological and sedimentological data reveal that terrestrial conditions in the region were in remarkable lockstep with the large-scale volcanism. Using the sedimentary mercury record as a proxy for eruptions reveals four discrete episodes during the CPE interval (ca. 234.0 to 232.4 Ma). Each eruptive phase correlated with large, negative C isotope excursions and major climatic changes to more humid conditions (marked by increased importance of hygrophytic plants), lake expansion, and eutrophication. Our results show that large igneous province eruptions can occur in multiple, discrete pulses, rather than showing a simple acme-and-decline history, and demonstrate their powerful ability to alter the global C cycle, cause climate change, and drive macroevolution, at least in the Triassic.

## Significance

The Carnian Stage of the Triassic Period marks one of the most significant intervals of the past 250 My. Within the space of ∼2 My, the world’s biota underwent major changes with dinosaurs becoming the notable incumbents. These events coincide with a remarkable interval of intense rainfall known as the Carnian Pluvial Episode (CPE). Here, we show, in a detailed record from a lake in North China, that the CPE can actually be resolved into four distinct events, each one driven by a discrete pulse of intense volcanism associated with enormous releases of carbon dioxide into the atmosphere. These triggered a major intensification of the hydrological cycle and led to lake eutrophication.

**Fig. 1.**
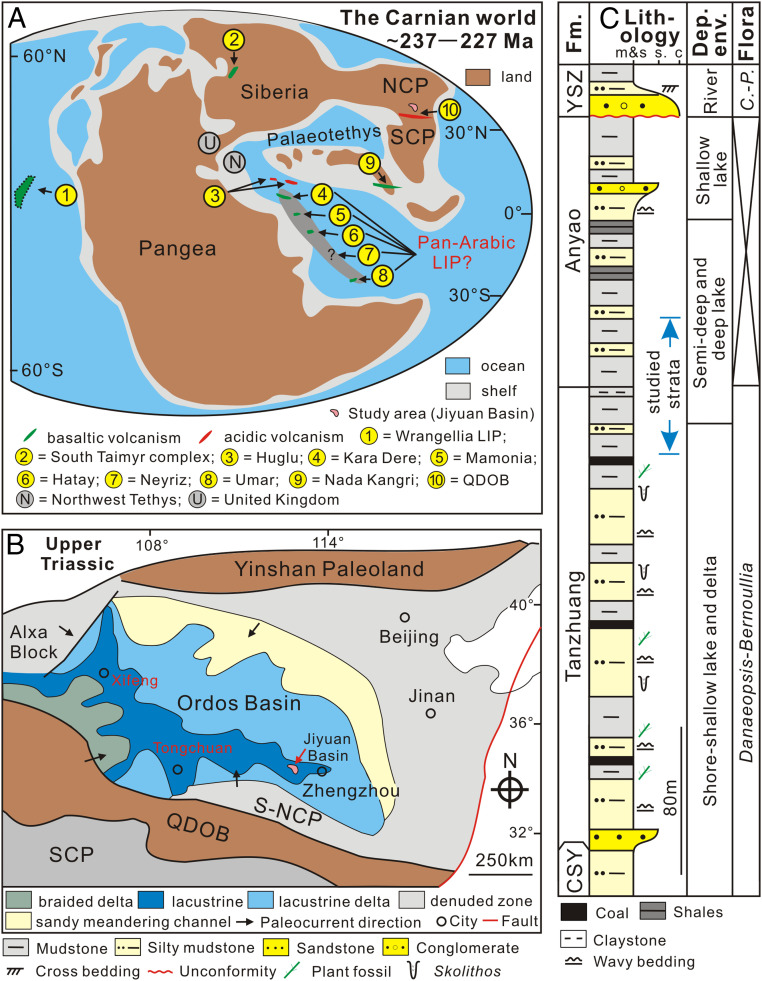
Location and geological context for the study area. (*A*) Paleogeographic reconstruction for the Carnian (∼237 to 227 Ma) Stage (Late Triassic), showing locations of the study area and volcanic centers (revised after ref. 4, with volcanic data from refs. 4, 7, 49, and 50). (*B*) Tectono-paleogeographic map of the NCP during the Late Triassic (modified from ref. 21), showing the location of the study area. (*C*) Stratigraphic framework of the Upper Chunshuyao Formation (CSY) to the Lower Yangshuzhuang (YSZ) Formation from the Jiyuan Basin (modified from ref. 20). Abbreviations: LIP, Large Igneous Province; QDOB, Qingling-Dabie Orogenic Belt; S-NCP, southern NCP; SCP, South China Plate; Fm., Formation; m & s, coal, mudstone, and silty mudstone; s., sandstone; c, conglomerate; Dep. env., Depositional environment; and *C*.-*P*., *Coniopteris*-*Phoenicopsis*.

The Carnian Pluvial Episode (CPE; ca. 234 to ∼232 Ma; Late Triassic) was an interval of significant changes in global climate and biotas (1, 2). It was characterized by warming (3, 4) and enhancement of the hydrological cycle (5–7), linked to repeated C isotope fluctuations (8–11) and accompanied by increased rainfall (1), intensified continental weathering (9, 12), shutdown of carbonate platforms (13), widespread marine anoxia (4), and substantial biological turnover (1, 2, 10). Available stratigraphic data indicate that the Carnian climatic changes broadly coincide with, and could have been driven by, the emplacement of the Wrangellia large igneous province (LIP) (2, 4, 7, 8, 10, 14, 15) (Fig. 1*A*). It is postulated that the voluminous emission of volcanic CO_2_, with consequent global warming and enhancement of a mega-monsoonal climate, was responsible for the CPE (9, 16), although the link is imprecise (2, 17) because the interval of Wrangellian eruptions have not yet been traced in the sedimentary records encompassing the CPE.

The CPE was originally identified because of changes in terrestrial sedimentation, but most subsequent studies have been on marine strata (2, 4, 7–10). By contrast, much less is known about the effects of this climatic episode on terrestrial environments (2), although there were major extinctions and radiations among animals (including dinosaurs, crocodiles, turtles, and the first mammals and insects) and modern conifer families (2). Some of the new organisms may have flourished because of the spread of humid environments, such as the turtles and metoposaurids (18, 19).

In this study, we have investigated terrestrial sediments from the Zuanjing-1 (ZJ-1) borehole in the Jiyuan Basin of the southern North China Plate (NCP) and use zircon U–Pb ages from two tuffaceous claystone horizons, fossil plant biostratigraphy, and organic C isotope (δ^13^C_org_) and Hg chemostratigraphy to identify the CPE and volcanic activity.

## Geological Setting

During the Triassic, the NCP was located in eastern Tethys, with the Yinshan Paleoland to the north and the Ordos Basin occupying the central and southwest areas of the continent (20–22). During the Early and Middle Triassic, the NCP was tectonically stable with clastic sedimentation occurring in fluvial–lacustrine settings in dry and hot climatic conditions. Later in the Triassic, the Qinling-Dabie Orogenic Belt (QDOB) formed during the collision and amalgamation of the NCP and South China Plate. This resulted in extensive volcanism (23, 24) and the migration of the Ordos Basin depocenter continuously westward (Fig. 1*B*) (21). The Jiyuan Basin was located in the southeast of the Ordos Basin (Fig. 1*B*) (21, 22) and was a lake of substantial depth that was receiving turbidite sediments from the Late Triassic into the Jurassic (22, 25). Sediment was mainly sourced from the QDOB and southern NCP (Fig. 1*B*) (20). The age of the Jiyuan succession is primarily constrained by its flora. The *Danaeopsis*–*Bernoullia* assemblage occurs in the Tanzhuang Formation, indicating a Late Triassic age (25), but the overlying Anyao Formation lacks biostratigraphically useful fossils and is tentatively assigned to the Early Jurassic based on its position between underlying Upper Triassic strata and the overlying Yangshuzhuang Formation. This unit contains fossil plants of the *Coniopteris*–*Phoenicopsis* floral assemblage, indicating a Middle Jurassic age (25, 26).

## Results

### Sedimentology.

The studied strata from the ZJ-1 core (345 to 205 m) record a change from shallow to deep lacustrine environment. Initially, the upper part of the Tanzhuang Formation (345 to 305 m; Stage I as defined by C isotope chemostratigraphy; see the *Chemostratigraphy* section) comprises silty mudstone, thin sandstone beds, and coal seams (*SI Appendix*, Fig. S1 *A* and *B*) with abundant plant fossils, vertical burrows (*SI Appendix*, Fig. S1 *C*–*G*), bivalves, conchostracans, and ostracods (25), suggesting shallow lake and swamp environments (25). Higher in the Formation (at ∼305 m), the development of laminated mudstone (*SI Appendix*, Figs. S1 *I*–*K*), turbidite sandstones (22, 27), and the disappearance of bioturbation indicates the deepening of the lake environments.

### U–Pb Zircon Dating.

Two tuffaceous claystones were sampled from the uppermost part of the Tanzhuang Formation; they are similar to other Late Triassic tuffs from the Ordos Basin (28). More than 2,000 zircon crystals were separated from the first sample in the Zuanjing-1 core from the Jiyuan Basin in Henan province (HJZ-1) and 1,500 from the second sample in the Zuanjing-1 core from the Jiyuan Basin in Henan province (HJZ-2), with crystal sizes varying from 50 to 200 µm. Most crystals show euhedral morphology and clear oscillatory zoning in cathodoluminescence (CL; *SI Appendix*, Fig. S2*A*). Th/U ratios from the zircon crystals vary from 0.49 to 2.39 [arithmetic mean (x̄) = 1.06; *SI Appendix*, Table S1]. Collectively, these features indicate that these are volcanic-sourced zircons (28).

**Fig. 2.**
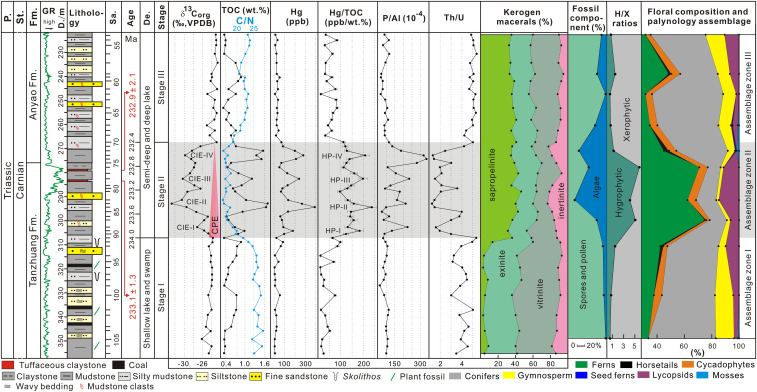
Results of zircon U–Pb dating, δ^13^C_org_ values, TOC contents, atomic C/N ratios, Hg concentrations, Hg/TOC ratios, P/Al and Th/U ratios, kerogen macerals, fossil component, hygrophytic/xerophytic (H/X) ratios, floral composition, and palynology assemblage from the studied borehole in the Jiyuan Basin. Note that error bars on Hg/TOC plots represent the propagated error on Hg (±5%) and TOC (±0.2%) content measurements. Abbreviations: P., Period; St., Stage; Fm., Formation; GR, natural gamma-ray curves; D., Deep; Sa., Sample; De., Depositional environment; VPDB, Vienna Pee Dee Belemnite; CIE-I to CIE-IV, from the first organic CIE to the fourth organic CIE; and HP-I to HP-IV, from the first Hg/TOC peak to the fourth Hg/TOC peak.

Results of zircon U–Pb laser ablation inductively coupled plasma mass spectrometry (ICP-MS) dating are shown in *SI Appendix*, Fig. S2 and Table S1. From sample HJZ-1, 42 concordant age values were distributed in a single peak, with a weighted mean ^206^Pb/^238^U age of 233.1 ± 1.3 Ma (mean-squared weighted deviation [MSWD] = 0.54, *n* = 42, and uncertainties are given at the 2σ level) (Fig. 2 and *SI Appendix*, Fig. S2*B*). Sample HJZ-2 yielded 18 concordant age values of which 11 were distributed in a single peak, with a weighted mean ^206^Pb/^238^U age of 232.9 ± 2.1 Ma (MSWD = 0.57, *n* = 11, and uncertainties are given at the 2σ level) (Fig. 2 and *SI Appendix*, Fig. S2*C*). The single-point analysis error of standard zircons Plešovice and 91500 is less than 2.2% (*SI Appendix*, Table S1). These ages indicate that the topmost Tanzhuang Formation belongs to the middle of the Carnian Stage (Fig. 2 and *SI Appendix*, Fig. S2) and are consistent with the Late Triassic age indicated by the spore–pollen assemblage (see results and analysis in *SI Appendix*).

### Chemostratigraphy.

The δ^13^C_org_ values vary from −32.7 to −22.8‰ (x̄ = −25.1‰; Fig. 2 and *SI Appendix*, Table S6). At the base of the succession (345 to 305 m), δ^13^C_org_ values are stable averaging around −24.5‰ (Stage I) (Fig. 2). From 305 to 279 m, beginning around the transition from shallow to deep water, there are a series of four negative δ^13^C_org_ excursions with values varying from −23.0 to −32.7‰ (x̄ = −27.4‰) (Stage II) and amplitudes that are −3.4‰ (C isotope excursions [CIE]–I), −7.8‰ (CIE-II), −3.8‰ (CIE-III), and −2.2‰ (CIE-IV) (Fig. 2). At the top of the succession (270 to 225 m), values return to heavier and stable values of ∼−23.7‰ (Stage III) (Fig. 2).

### Palynology and Macerals.

**Fig. 3.**
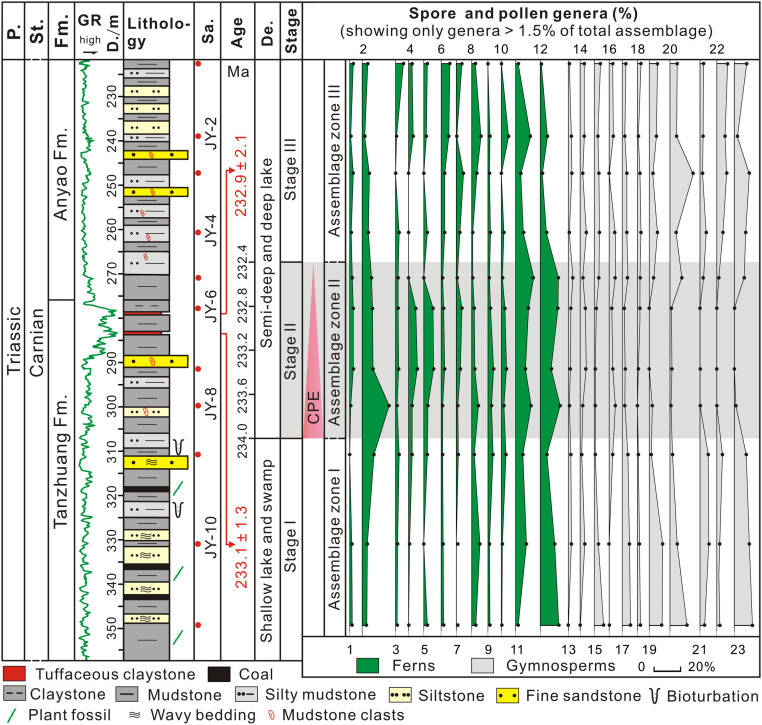
Results of spore–pollen species from the studied borehole in the Jiyuan Basin. Note that spore–pollen species of more than 1.5% abundance are plotted. More detailed data are shown in *SI Appendix*, Table S2. Abbreviations: P., Period; St., Stage; Fm., Formation; GR, natural gamma-ray curves; D., Deep; Sa., Sample; De., Depositional environment; 1, *Baculatisporites*; 2, *Osmundacidites*; 3, *Crassispora*; 4, *Kraeuselisporites*; 5, *Triquitrites*; 6, *Lophotriletes*; 7, *Anapiculatisporites*; 8, *Verrucosisporites*; 9, *Cyathidites*; 10, *Laevigatosporites*; 11, *Punctatisporites*; 12, *Cyclogranisporites*; 13, *Cycadopites*; 14, *Chasmatosporites*; 15, *Psophosphaera*; 16, *Inaperturopollenites*; 17, *Vesicaspora*; 18, *Cordaitina*; 19, *Rotundipollis*; 20, *Pseudopicea*; 21, *Protopinus*; 22, *Protoconiferus*; and 23, *Paleoconiferus*.

A total of 26 genera of spores, 28 genera of pollen, and 5 genera of algae have been identified; they contain many taxa typical of the Late Triassic (Fig. 3 and *SI Appendix*, Fig. S3 and Table S2) and are assigned to three palynological assemblage zones: the *Paleoconiferus*–*Cyclogranisporites*–*Rotundipollis* assemblage zone (AZ-I, samples #JY 11 to #JY 9), the *Cyclogranisporites*–*Osmundacidites*–*Punctatisporites* assemblage zone (AZ-II, sampled #JY 8 to #JY 5), and the *Pseudopicea*–*Paleoconiferus*–*Protoconiferus* assemblage zone (AZ-III, samples #JY 4 to #JY 1). In AZ-I to AZ-III, gymnosperm pollen typically belong to the conifer families Pinaceae, Podocarpaceae, and Taxodiaceae and to cycads, while fern spores belong to the families Dipteraceae, Cyathcaceae, and Osmundaceae as well as members of the Selaginellaceae from the Lycopsida (Fig. 2 and *SI Appendix*, Table S3). In modern flora, these families mainly grow in temperate to subtropical, warm, and humid climates (29). The compositions of AZ-I and AZ-III are broadly similar: Gymnosperm pollen dominate (x̄ = 52.3 and 47.6%, respectively) and include *Paleoconiferus* and *Pseudopicea*, followed by fern spores, including *Cyclogranisporites*, *Osmundacidites*, *Punctatisporites*, and a few algae (Fig. 3 and *SI Appendix*, Table S2). In contrast, in AZ-II, algae dominate (x̄ = 56.5%, including *Leiosphaeridia*, *Granodiscus*, *Verrucosphaera tuberculata*, and *Micrhystridium*) together with fern spores (x̄ = 33.3%, including *Cyclogranisporites*, *Osmundacidites*, and *Punctatisporites*), while gymnosperm pollen are less abundant (x̄ = 10.2%, including *Inaperturopollenites*, *Pseudopicea*, and *Chasmatosporites*) (Figs. 2 and 3 and *SI Appendix*, Table S2). Compared with AZ-I and AZ-III, hygrophytic plants, including all spores and pollen of *Cycadopites*, increased in AZ-II, and this zone also includes the only record of the spore *Alisporites* in our samples (Figs. 2 and 3 and *SI Appendix*, Table S2), as well as hygrophytic/xerophytic ratios (Fig. 2; see results and analysis in *SI Appendix*), all indicating an intensification of humidity (6, 30). An increase of spores relative to pollen abundance, with an increase in the importance of *Alisporites*, is also recorded in the Carnian terrestrial Dunscombe Mudstone in southwest England, where it coincides with a rise of freshwater algae; these changes are interpreted as evidence for lake expansion during the CPE (30). The three palynological assemblage zones AZ-I to AZ-III, correspond broadly with three C isotope stages, although the four negative excursions in Stage II do not coincide with any palynomorph fluctuations, indicating that the CIEs are not the result of organic matter (OM) variations.

Vitrinite content varies from 13.4 to 55.1% (x̄ = 30.7%) (*SI Appendix*, Table S4) and comprises nonfluorescent telinite and collinite (Fig. 2 and *SI Appendix*, Fig. S4 *A* and *B*). Exinite content varies from 18.9 to 42.4% (x̄ = 30.0%) and mainly comprises sporopollenite, cutinite, and suberinite (Fig. 2 and *SI Appendix*, Fig. S4 *C* and *D*). Sapropelinite content varies from 1.9 to 43.2% (x̄ = 26.0%) (Fig. 2 and *SI Appendix*, Fig. S4*E*). Inertinite content varies from 5.2 to 21.3% (x̄ = 13.4%) and is entirely fragmental fusinite (*SI Appendix*, Fig. S4*F*). *T*_max_ values vary from 441 to 454 °C (x̄ = 445.6 °C) (*SI Appendix*, Table S4) and indicate that kerogen maturity varies from low maturity to mature (*SI Appendix*, Table S5).

Total organic C (TOC) varies from 0.32 to 1.70 weight percentage (wt%) (x̄ = 0.70 wt%), and C/N ratios vary from 15.79 to 27.76 (x̄ = 21.38) (Fig. 2 and *SI Appendix*, Table S6). The lower and upper part of the sedimentary succession (roughly corresponding to Stages I and III, respectively) have relatively low-TOC values ranging from 0.34 to 0.75 wt% (x̄ = 0.53 wt%) and 0.32 to 0.87 wt% (x̄ = 0.55 wt%), respectively, and relatively high-C/N ratios, ranging from 21.83 to 27.76 (x̄ = 25.53) and 19.16 to 23.86 (x̄ = 22.19), respectively. TOC values in the middle (roughly corresponding to C isotope Stage II) are relatively high, ranging from 0.34 to 1.70 wt% (x̄ = 0.93 wt%), with relatively lower-C/N ratios, ranging from 15.79 to 19.90 (x̄ = 17.37) (*SI Appendix*, Table S6).

### P/Al and Th/U Ratios.

Results of P/Al and Th/U ratios are shown in Fig. 2 and *SI Appendix*, Table S6. P/Al varies from 67.3 × 10^−4^ to 511.3 × 10^−4^ (x̄ = 120.2 × 10^−4^) (Fig. 2) and has a vertical distribution showing two relatively stable intervals of low values (Stages I and III) and an interval with enrichments (Stage II) (Fig. 2). The latter consists of four P/Al peaks (from the bottom to the top: 217.26 × 10^−4^, 178.44 × 10^−4^, 234.15 × 10^−4^, and 511.28 × 10^−4^) that correspond to the four negative CIEs (Fig. 2).

Th/U ratios vary from 1.24 to 5.37 (x̄ = 3.75) (Fig. 2) and have a vertical distribution pattern that is similar to δ^13^C_org_ and shows two relatively stable, high-value intervals separated by a more strongly fluctuating, low-value interval in Stage II. Thus, there are four extreme values of Th/U ratio, with values from the bottom to the top of 1.6, 1.24, 1.52, and 1.31 that correspond to four negative δ^13^C_org_ excursions (Fig. 2).

### Hg Geochemistry.

Hg concentrations show considerable variation, ranging from 5 to 391 ppb (x̄ = 71.52 ppb) and have a distribution that is broadly anticorrelated with the δ^13^C_org_ values (Fig. 2 and *SI Appendix*, Table S6). Highest-Hg values occur during C isotope Stage II (Fig. 2). The Hg concentrations show stronger covariation with TOC (r = +0.89, *n* = 55) than with Al (r = +0.33) or total sulfide (TS; r = +0.38) (*SI Appendix*, Fig. S5), suggesting that Hg is mostly hosted by OM. Therefore, we present Hg/TOC values to evaluate Hg concentration enrichments. These vary from 10.79 to 236.97 ppb/wt% (x̄ = 84.16 ppb/wt%) and show a pattern similar to the Hg concentrations (Fig. 2): two relatively stable intervals with low values corresponding to C isotope Stages I and III and an interval with higher values that includes four anomalies that coincide with the CIEs within Stage II (Fig. 2 and *SI Appendix*, Fig. S6).

## Discussion

### Identification of the CPE in the Nonmarine Jiyuan Basin.

New U–Pb ages, chemostratigraphy, and palynology constrain the CPE interval in the studied basin and allow correlation with the marine reference successions.

Based on cyclostratigraphy, biostratigraphy, and magnetostratigraphy evidence, previous studies have determined that the CPE occurred between ca. 234 and 232 Ma and lasted ∼1.2 to 1.7 Ma from the late Julian 1 to the Tuvalian 2 substages of the Carnian (2, 11, 31, 32). In the study area, the AZ-I–AZ-III sporomorph assemblages contain typical Late Triassic elements (*SI Appendix*), and the new zircon U–Pb ages of 233.1 ± 1.3 Ma and 232.9 ± 2.1 Ma, from the top of the Tanzhuang Formation, lie within the age limits of the CPE (Fig. 2).

**Fig. 4.**
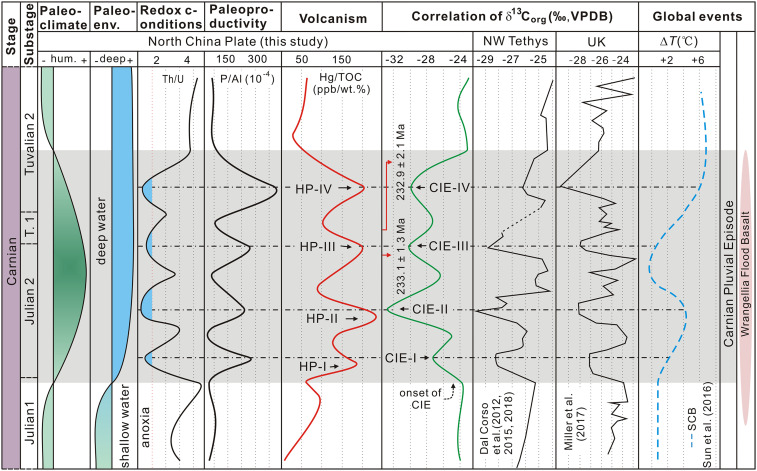
Correlations of paleoclimate, paleoenvironment, redox conditions, volcanism, C isotope records, and global events from Julian 1 to Tuvalian 2 Substages during the Carnian Stage. The stratigraphic framework is from ref. 2. Abbreviations: T. 1, Tuvalian 1; hum., humidity; Paleoenv., paleoenvironment; CIE-I to CIE-IV, organic CIEs I to IV; HP-I to HP-IV, Hg/TOC peak I to IV; VPDB, Vienna Pee Dee Belemnite; NW, Northwest; UK, United Kingdom; and SCB, South China Block.

C isotope stratigraphy shows multiple sharp negative CIEs (CIE-I to CIE-IV) within the studied succession (C isotope Stage II; Fig. 2). Rock-Eval pyrolysis indicates that OM from the CIE-I to CIE-IV interval in the Jiyuan Basin varies from low maturity to mature with respect to oil generation (*SI Appendix*, Tables S4 and S5), but diagenetic processes are unlikely to have produced large changes in the δ^13^C_org_ signature because these occur in late diagenetic to metamorphic stages (9, 33). The CIEs are also unlikely to have resulted from proportional changes in terrestrial versus algal organic C sources, which can be isotopically distinct from one another (34). Variations in kerogen macerals reveal that the OM in Stage II was from mixed sources of terrestrial plants and lacustrine plankton (see results and analysis in *SI Appendix*). However, no changes of maceral composition and palynomorph abundance are recorded during the CIEs. Furthermore, relatively high-C/N ratios suggest that the OM throughout the succession is predominantly terrestrial with lower-C/N ratios supporting a proportional increase in algal production during Stage II (34, 35) (*SI Appendix*), but crucially, there is no apparent relationship between C/N and δ^13^C across the CIEs. Thus, the multiple negative CIEs recorded in the Jiyuan succession are interpreted as the global anomalies associated with the CPE (Fig. 4). Indeed, previous studies have found multiple CIEs during the CPE interval, including three to four significant negative excursions recorded by bulk OM, biomarkers, and marine carbonates in western Tethyan marine sequences [e.g., Italy and Hungary (2, 10)], South China and Oman (4, 7), continental sequences from the United Kingdom (2, 10, 11, 30), and deep-water successions of Panthalassa (Japan) (15) (Fig. 4).

The first negative CIE (∼2 to 4‰) at the boundary between the Julian 1 and 2 marks the onset of the CPE (2, 6, 10, 11) and coincides with an increase of terrestrial hygrophytes (6). These features are similar to those of CIE-I (−3.4‰) in the study area and the development of wetter climates indicated by spore–pollen assemblages, which mark the bottom of our Stage II (Figs. 2 and 4). The CIE-I in the Jiyuan succession is followed by three negative CIEs, as seen in the marine and terrestrial C isotope records from other areas (Fig. 4). The boundary of C isotope Stages II and III in the Jiyuan Basin is defined by the positive rebound of the last recorded negative CIE (CIE-IV), which is followed by a relatively stable, isotopic plateau (Stage III; Figs. 2 and 4), a transition we take to represent the end of the CPE. Extrapolating the sedimentation rate between the two U–Pb ages (25 m/Ma) to the entire succession yields an approximate duration for C isotope Stage II of 1.6 Ma (Fig. 2), with the onset at ∼234.0 Ma and the end at ∼232.4 Ma. Thus, the onset and duration of Stage II in the Jiyuan Basin is close to estimates for the duration of the CPE elsewhere.

### CPE-Related Lacustrine Environmental Changes.

Warming and increased evaporation during the CPE likely resulted in the enhancement of the global atmospheric circulation and the hydrological cycle, causing the widespread transition from dry to more humid conditions and a consequent increase of continental runoff (2, 4, 7) (Fig. 4 and *SI Appendix*, Fig. S7).

Spore and pollen assemblages in the Jiyuan Basin of the larger Ordos Basin are all dominated by hygrophytic fern spores throughout the Carnian, indicating a general warm and humid climate in the area, but an increase of hygrophytic elements in AZ-II (C isotope Stage II), including all spores, *Alisporites*, and *Cycadopites*, indicate an intensification of humid conditions in this region (Figs. 2 and 3). This is coeval with the increase of hygrophytic plants observed in western Tethys during the CPE (5, 6, 36), in which herbaceous filicalean ferns, Lycopodiales (clubmosses), Equisetopsida (horsetails), and Cycadeoids (2, 5, 6) proliferated, indicating the widespread intensification of the hydrological cycle.

The increase of humidity, associated with the lake deepening in the Jiyuan Basin (Figs. 2 and 3), is also seen in coeval successions across Pangea, including the Central European Basin (1), Wessex Basin (United Kingdom) (30), East Greenland (37), Morocco (38), Newark Basin [North America (39)], and the Ischigualasto Basin [Argentina (17, 40)], indicating that increased rainfall resulted in the widespread expansion of the endorheic basins during the CPE (2).

Higher humidity also intensified runoff and nutrient input in the Jiyuan Basin, leading to the development of dysoxic conditions indicated by decreased Th/U values (41) the loss of bioturbation, and a bloom of planktonic algae (C/N ratios; Fig. 2), all suggesting eutrophication occurred during the CPE (Figs. 2 and 3 and *SI Appendix*, Fig. S7). Such changes may have been important negative feedbacks in the global C cycle by increasing the sequestration of organic C in lacustrine settings, as also observed during the Toarcian Oceanic Anoxic Event (42). Moreover, the eutrophic Jiyuan lake lasted for the entire duration of the CPE (ca. 1.6 Ma), indicating a prolonged impact on lacustrine systems that was a component of substantial and diverse environmental changes during the CPE.

### A Volcanic Driver of the CPE.

The eruption of the Wrangellia LIP is commonly regarded as the cause of the CPE (2, 4, 7, 14). In the Jiyuan Basin, C isotope Stage II and associated environmental changes are synchronous with generally higher-Hg concentrations and Hg/TOC levels with respect to background levels (Stages I and III) (*SI Appendix*, Fig. S6), with their multiple peaks closely coinciding with the negative CIEs (Fig. 2). Hg in lakes can arrive from riverine influx or via direct atmospheric deposition (43, 44). As the principal host of Hg in this study (see *Results*), OM derives from a mixture of terrestrial higher plants and lacustrine plankton in Stage II. Minor increases in TOC pre- and post-CPE in Stages I and III are not accompanied by Hg peaks (Fig. 2), supporting the notion that terrestrial OM within Stage II was enriched in Hg because of volcanism in the environment before flux to the lake. We conclude that Hg/TOC data from the Jiyuan Basin are reliable indicators of intense volcanic activity during the CPE: Four distinct episodes of enhanced Hg flux into the lacustrine environment were linked to CIEs and episodes of environmental perturbation. While it is possible that there is a local volcanic source for Hg, as supported by the presence of two tuff beds (Fig. 2 and *SI Appendix*, Fig. S2), it is likely that the Hg enrichment comes from a major source of volcanism that was capable of impacting the global C cycle. The obvious candidate is Wrangellia, implying that the eruptions of this LIP occurred in four major pulses.

Volcanic activity can release a large amount of CO_2_ into the ocean–atmosphere system and trigger global warming (2, 4). However, it is unclear if the Wrangellia eruptions could have supplied sufficient C to cause the four observed negative CIEs directly or whether C sources from positive feedbacks (e.g., methane hydrate release or the transfer of organic C from the lithosphere or biosphere into the ocean–atmosphere system) are required. Conservative estimates suggest that Wrangellia emplacement released ∼5 × 10^3^ Pg of mantle C (8). Additional C emissions through contact metamorphism are unlikely because of the oceanic site of eruption (45). Using cGENIE, an intermediate complexity Earth system model, Vervoort et al., use a large ensemble of experiments to constrain 1) the C emissions required to generate negative CIEs of different sizes, durations, and C sources; 2) the C removal fluxes required to subsequently restore δ^13^C; and 3) the environmental effects of each emissions scenario. These simulations suggest a negative CIE of ≥3‰, lasting ∼10 to 100 s of thousands of years (kyr) (of which the CPE hosts four), requires in excess of 3 × 10^4^ PgC, if the mantle source of C has a δ^13^C value of −6‰ (46). These model simulations do not include the isotopic effects of concurrent TOC burial (Fig. 2), which renders the estimate of required C emissions highly conservative. Furthermore, each of the negative CIEs during the CPE is followed by a subsequent “recovery.” If the source C is a relatively enriched mantle C (−6‰) and the recovery is driven only by organic C burial (−22‰), then these same model simulations suggest each CIE recovery requires a burial of ∼10^4^ PgC, which is several times the C storage of the entire modern terrestrial biosphere (46). While black shales are quite extensive during the CPE (2, 4, 7), this is not to the extent seen during the intervals of ocean anoxia (e.g., in the Early Triassic and Late Cretaceous), thereby making the C drawdown mechanism somewhat enigmatic. However, if the source C is more isotopically depleted, each CIE recovery would require more modest C drawdown. Potentially, volcanic C from the mantle plume that supplied the Wrangellia LIP could be isotopically lighter, or the CIE could have been partly driven by positive C cycle feedbacks [e.g., terrestrial or rock-bound organic C oxidation (47) or methane release from hydrate reservoirs].

Despite the generally low resolution of available conodont O isotope data, available evidence indicates that the CPE was a global warming event. Thus, O isotope data, from somewhat incomplete sections in the Northern Calcareous Alps and the Lagonegro Basin, records warming from the late Julian to the Tuvalian of about 6 to ∼8 °C (3, 48). Higher-resolution O isotope data from the Nanpanjiang Basin records two warming events in the Julian 2 substage and Tuvalian 1 substage (∼4 and ∼6 °C) (2, 4) (Fig. 4). Given the size and duration of the negative CIEs, a 4 to 8 °C warming is consistent with a depleted (−22‰, organic C) or intermediate C source (−12‰; e.g., volcanism plus isotopically depleted C released through positive C cycle feedbacks) and inconsistent with a predominant mantle source with a canonical −6‰ composition (46).

In conclusion, our high-resolution study shows that the four pulses of LIP volcanism were likely responsible for the global negative CIEs that mark the CPE and drove major environmental changes in the lacustrine Jiyuan Basin of North China, including more humid conditions and lake expansion and eutrophication. The consequences of this relatively long (ca. 1.6 Ma) interval of volcanism and climate and environmental changes on land included the diversification of dinosaurs and modern conifer groups and overall saw a major impact on the evolutionary direction of Mesozoic terrestrial biota.

## Materials and Methods

Two gray-white, tuffaceous claystones (sample HJZ-1 and HJZ-2) were collected from the uppermost part of the Tanzhuang Formation in the ZJ-1 borehole (35.07001° N, 112.47338° E) of the Jiyuan Basin (Fig. 2 and *SI Appendix*, Fig. S2). Zircons were separated for U–Pb dating. In addition, 55 fresh mudstone samples were collected from the Tanzhuang and Anyao formations for geochemical and palynological analyses after eliminating drilling mud contamination (Figs. 2 and 3).

### Geochronology.

From each tuffaceous claystone bed, ∼l kg material was processed for zircon separation; this yielded abundant crystals (*SI Appendix*, Fig. S2*A*). After crushing, grinding, sieving, and heavy liquid and magnetic separation, euhedral zircon crystals, with clear oscillatory zoning under CL microscope, were selected for U–Pb zircon isotope analysis. U–Pb dating was conducted at the State Key Laboratory Geological Processes and Mineral Resources (Beijing) using a Thermo Fisher Scientific X-Series 2 ICP-MS instrument to acquire ion signal intensities. Laser sampling was performed using a Coherent GeoLasPro193-nm system. Zircon 91500 and Plešovice zircon were used as an external standard for U–Th–Pb isotopic ratios and monitoring the standard of each analysis, respectively. Data Cal and Isoplot 3.0 software were used for the age analysis, calculation, and the drawing of concordia diagrams from the ICP-MS data.

### Geochemistry.

Each sample was crushed below the 200 mesh and divided into six subparts for 1) δ^13^C_org_ analysis, 2) TOC analysis, 3) major elements analysis, 4) trace elements analysis, 5) Hg content analysis, and 6) TS analysis. Hg concentrations were measured at the State Key Laboratory of Coal Resources and Safe Mining (Beijing), while δ^13^C_org_, TOC, major and trace elements, and TS were measured at the Beijing Research Institute of Uranium Geology. δ^13^C_org_ analysis was performed using a stable isotope mass spectrometer (MAT253), and δ^13^C_org_ values are expressed in per mil (‰), with respect to the Vienna Pee Dee Belemnite standard, with an absolute analysis error of ± 0.1‰. Samples for TOC were first treated with phosphoric acid to remove inorganic C, and then the TOC values were measured using a C–S analyzer (Eltra CS580-A) with the lower-detection limits of 100 μg/g and the absolute analytical error of ± 0.2%. Major elements analysis was undertaken with an X-ray fluorescence spectrometer (PW2404) with the relative analytical error of ± 5%. Trace elements analysis was undertaken using an inductively coupled plasma mass spectrometer (Element XR) with the relative analytical error better than ± 5%. TS analysis was performed using a C–S analyzer (Eltra CS580-A) with the lower-detection limits of 30 ppm and the absolute analytical error of ± 5%. Hg concentration was undertaken using a Hg analyzer (Lumex RA-915+) with lower-detection limits of 2 ng/g (2 ppb) and the relative analytical error of ± 5%. The relative or absolute error of all samples is based on reproducibility and repeats of the special standard sample and standard run after every five sample analyses.

### Kerogen Macerals.

Kerogen enrichment and identification were undertaken on 30 out of 55 mudstone samples (Fig. 2), according to the China national standard (SY/T5125–2014) at the Research Institute of Petroleum Exploration and Development Research (Beijing). At least 300 effective points per sample were analyzed by the point-counting method under a microscope.

### Rock-Eval Pyrolysis.

Rock-Eval pyrolysis was undertaken from 10 selected mudstones samples distributed vertically in the succession (*SI Appendix*, Table S4). Samples were analyzed using a second generation oil and gas evaluation (OEG-II) workstation, according to the China National Standard (GB/T18602-2012) at the Research Institute of Petroleum Exploration and Development Research (Beijing).

### Palynology.

Palynological isolation and identification were undertaken from 11 out of 55 mudstone samples (Figs. 2 and 3). Samples were first crushed into particles less than 1 mm in diameter before acid digestion in 30% hydrochloric acid (HCl) and 38% hydrofluoric acid (HF) and heavy mineral separation to concentrate the sporomorphs and separate them from other components of the residue. For each spore–pollen sample, more than 100 sporomorphs were identified by the point-counting method under a transmitted light microscopy (Olympus BX 41).

## Supplementary Material

Supplementary File

## Data Availability

The core, palynological slides and zircon samples are housed at the State Key Laboratory of Coal Resources and Safe Mining (Beijing). All other study data are included in the article and/or *SI Appendix*.

## Change History

October 8, 2021: The license for this article has been updated.
