# Supplementary material for: Volcanically driven lacustrine ecosystem changes during the Carnian Pluvial Episode (Late Triassic)
Source: Proc Natl Acad Sci U S A. 2021 Sep 27;118(40):e2109895118. doi: 10.1073/pnas.2109895118 (PMC8501800; doi:10.1073/pnas.2109895118)
Supplement: Supplementary File [file pnas.2109895118.sapp.pdf]

Supporting Information Appendix

Volcanically driven lacustrine ecosystem changes during the Carnian Pluvial Episode (Late Triassic)

Jing Lu, Peixin Zhang, Jacopo Dal Corso, Minfang Yang, Paul B. Wignall, Sarah E. Greene, Longyi Shao, Dan Lyu, Jason Hilton*

*Corresponding Author. Email: j.m.hilton@bham.ac.uk

- SUPPLEMENTARY TEXT 2
  - Geochronology 2
  - Geochemistry 2
  - Hygrophytic, xerophytic floral elements 2
  - Kerogen macerals and C/N ratios 2
  - Stratigraphic correlation 3
- SUPPLEMENTARY FIGURES 4
- SUPPLEMENTARY TABLES 9
- LITERATURE CITED (for Supporting Information only) 19

Supplementary Text

Geochronology

U-Pb dating was conducted at the Ore Deposit Geochemistry Microanalysis Laboratory, affiliated to the State Key Laboratory of Geological Processes and Mineral Resources, China University of Geosciences. Laser sampling was performed using a Coherent's GeoLasPro-193nm system. A Thermo Fisher X-Series 2 ICP-MS instrument was used to acquire ion-signal intensities. Helium was applied as a carrier gas. Argon was used as the make-up gas and mixed with the carrier gas. All data were acquired on zircons in single spot ablation mode at a spot size of 32 μm with 6 Hz frequency in this study. Standard material SRM610 from National Institute of Standards and Technology (NIST) of America was used to optimize the ICP-MS instrument, and as an external standard for determination of trace elements. Zircon 91500 was used as an external standard for U-Th-Pb isotopic ratios (1, 2). Plešovice Zircon was used as a monitoring standard for each analysis (3). Time-dependent drifts of U-Th-Pb isotopic ratios were corrected using a linear interpolation (with time) for every five analyses according to the variations of 91500 (i.e., 2 zircon 91500 + 5 samples + 2 zircon 91500). The result of our study shows that the single point analysis of Plešovice zircon and 91500 is less than 2.2% (Table S1). Each analysis incorporated a background acquisition of approximately 20s (gas blank) followed by 50s data acquisition from the sample. Off-line selection and integration of background and analyte signals, and time-drift correction and quantitative calibration for trace element analyses and U-Pb dating were performed by ICPMSDataCal (4). Data reduction and concordia diagram was carried out using the Isoplot 3.0 (5).

Geochemistry

Atomic C/N ratios analysis

According to the China National Standard (GB/T19143-2017; Analytical method of elements for carbon, hydrogen, oxygen, and nitrogen in rock organics), when TOC content is less than 3% and/or Loss-on ignition (LOI) is less than 75%, it is essential to enrich OM before evaluating the C and N element content. Before enriching the OM, we used HCl and HF to remove carbonate and silicate respectively. After this, the C and N element contents were analyzed using an elemental analyzer (vario MICRO Cube) according to the China National Standard (GB/T19143-2017) at the Research Institute of Petroleum Exploration and Development Research (Beijing). To quantify the analytical results, a certified reference material (L-alanine) was used during the analysis. Analytical errors were 0.05% for C and N element contents.

Hygrophytic, xerophytic floral elements

According to the climate affinity of the palynological fossils, the identified spores and pollens were classified into hygrophytes and xerophytes. A hygrophytic/xerophytic (H/X) ratio is a first-order approximation of a humidity signal, unless any of the exceptions mentioned occur in high abundance (6, 7). Following the classification of previous studies that all spores are classified as hygrophytes together with the *Alisporites*, *Aulisporites* and *Cycadopites* groups, and all remaining pollen are classified as xerophytic (6, 7). Results of H/X ratios are shown in table S2. H/X varies from 0.57 to 5.81 ($\bar{x}$ = 2.05) and increased in Stage II (Fig. 2).

Kerogen macerals and C/N ratios

The vitrinite, inertinite, and exinite groups are derived principally from terrestrial higher plants, whereas the sapropelinite group is derived from lacustrine plankton, predominantly algae (8). In the study area, Stage I strata are mainly shallow lake and swamp sediments, and organic matter (OM) is mainly vitrinite ($\bar{x}$ = 45.0%), exinite ($\bar{x}$ = 37.9%) and inertinite ($\bar{x}$ = 11.6%) derived from in-situ higher plants (Fig. 2). Stages II and III strata are mainly semi-deep and deep lake sediments, and the OM are mainly sapropelinite group ($\bar{x}$ = 38.9% and 29.1%, respectively) formed by algae, followed by vitrinite ($\bar{x}$ = 20.5% and 29.9%, respectively), exinite ($\bar{x}$ = 23.4% and 30.8%, respectively) and

inertinite (x̄ = 17.2% and 10.2%, respectively) groups from terrestrial plants (Fig. 2).

C/N ratios have been used to determine the provenance of sedimentary OM from the lacustrine environment (9–12). Previous studies have shown that the increase in C/N ratios indicates a higher contribution of terrestrial OM, whereas the decrease in C/N ratios indicates a higher contribution of aquatic OM (9–12). In this study, the results of C/N ratios are shown in table S6. Atomic C/N values varies from 15.79 to 27.76 (x̄ = 21.38), with two relatively stable intervals of high values (Stages I and III) and an interval with relatively low values (Stage II). In Stages I and III, atomic C/N ratios varies from 21.83 to 27.76 (x̄ = 25.53) and 19.16 to 23.86 (x̄ = 22.19) respectively (table S6), consistent with OM that is predominantly terrestrial plants; modern vascular land plants have C/N ratios of 20 and greater (9–11). In Stage II, atomic C/N ratios varies from 15.79 to 19.90 (x̄ = 17.37) (table S6), likely reflecting an increased contribution of phytoplankton, which typically have atomic C/N ratios between 4 and 10 (9–11).

Stratigraphic correlation

The spore-pollen data indicate that the Jiyuan Basin strata (upper part of Tanzhuang Formation and the lower part of Anyao Formation) are of Carnian (Late Triassic) age.

The spore-pollen fossils from AZ-I to AZ-III contain typical Late Triassic elements including *Dictyophyllidites*, *Aratrisporites*, *Asseretospora*, *Cadargasporites*, *Calamospora*, *Kraeuselisporites*, *Punctatisporites*, and *Laevigatosporites* (13–18), as well as sporadic *Chordasporites* (fig. S3), *Caytonipollenites* (fig. S3), and *Taeniaesporites*. Similarly, the assemblages also contain *Caytonipollenites* (fig. S3), *Piceaepollenites* (fig. S3), *Podocarpidites* (fig. S3), *Cycadopites* (fig.S3), *Chasmatosporites* (fig. S3), and *Psophosphaera* (fig. S3) which are characteristic of the Late Triassic pollen assemblages. Furthermore, these three assemblages contain a limited number of Jurassic pioneer taxa such as *Cythidites*, *Quadraeculina*, *Cerebropollenites*, and *Perinopollenites* (19), Thus, combined with the zircon U-Pb ages of the tuffaceous claystones, the Jiyuan Basin spore-pollen assemblages can be considered of Carnian age.

Supplementary Figures

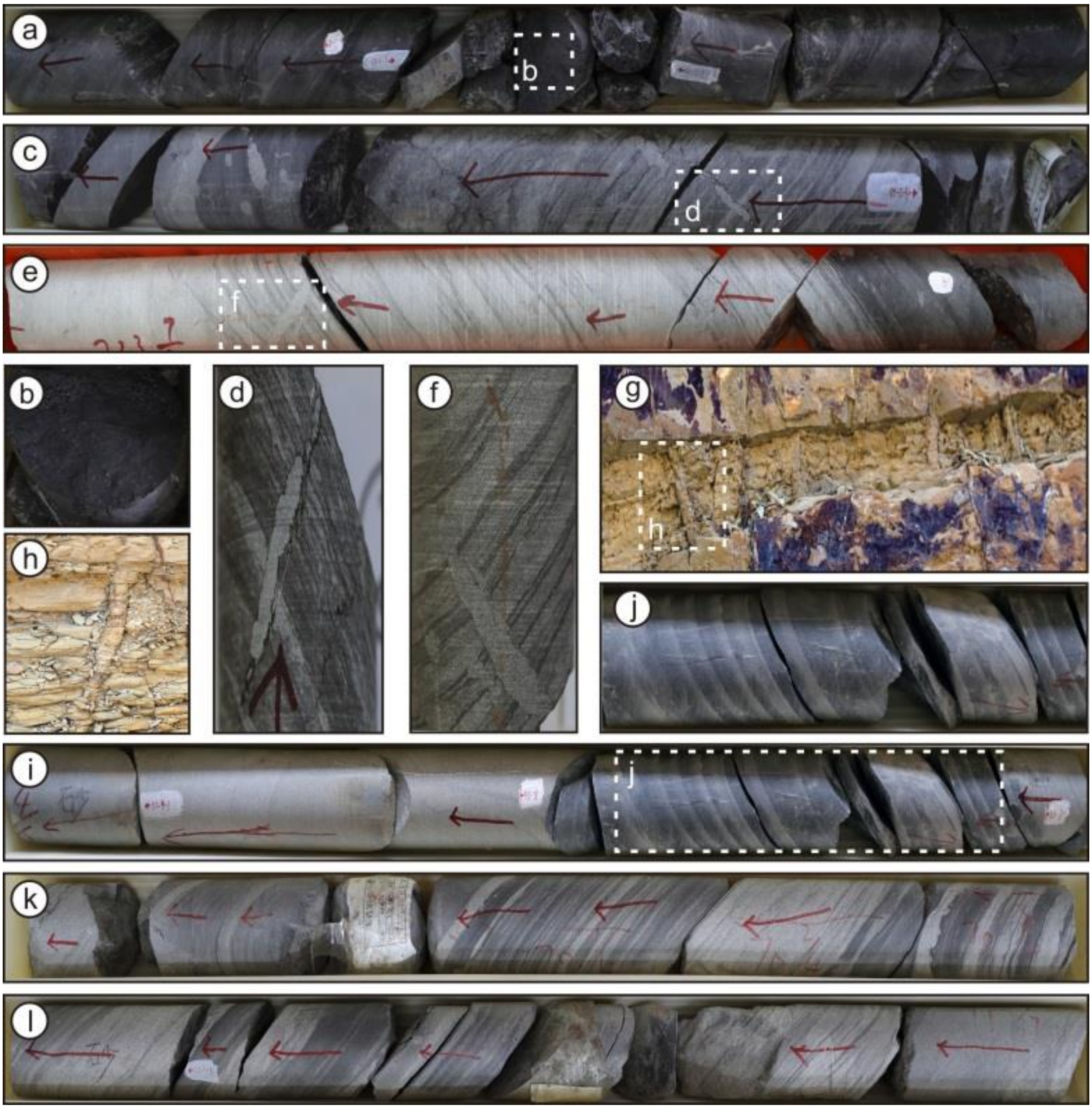

Figure S1. Photographs of the studied borehole and Sanhuang Village section (near the studied borehole) in the Jiyuan Basin showing features of the coals, bioturbation and sedimentary structures. **a** and **b**, The Tanzhuang Formation in the studied borehole showing features of the coal. **c**, **d**, **e**, and **f**, Photographs from the Tanzhuang Formation in the studied borehole showing features of the the bioturbation. **g** and **h**, Photographs from the Tanzhuang Formation at Sanhuang showing *Skolithos*. **i**, **j**, **k** and **l**, Photographs from the Upper Tanzhuang Formation and the lower Anyao Formation showing undisturbed thin bedding and fine lamination. Note photographs **a-g** shows the shoreline, shallow lake and delta environments in the upper part of Tanzhuang Formation (345–205 m), photographs **i-l** shows the deeper lake environments in the higher parts of the Tanzhuang Formation (at ~305 m). Sedimentary structures help determine the oxygenation of the CPE events, with fine lamination (20) and lack of trace fossils indicating dysoxic or anoxic environments.

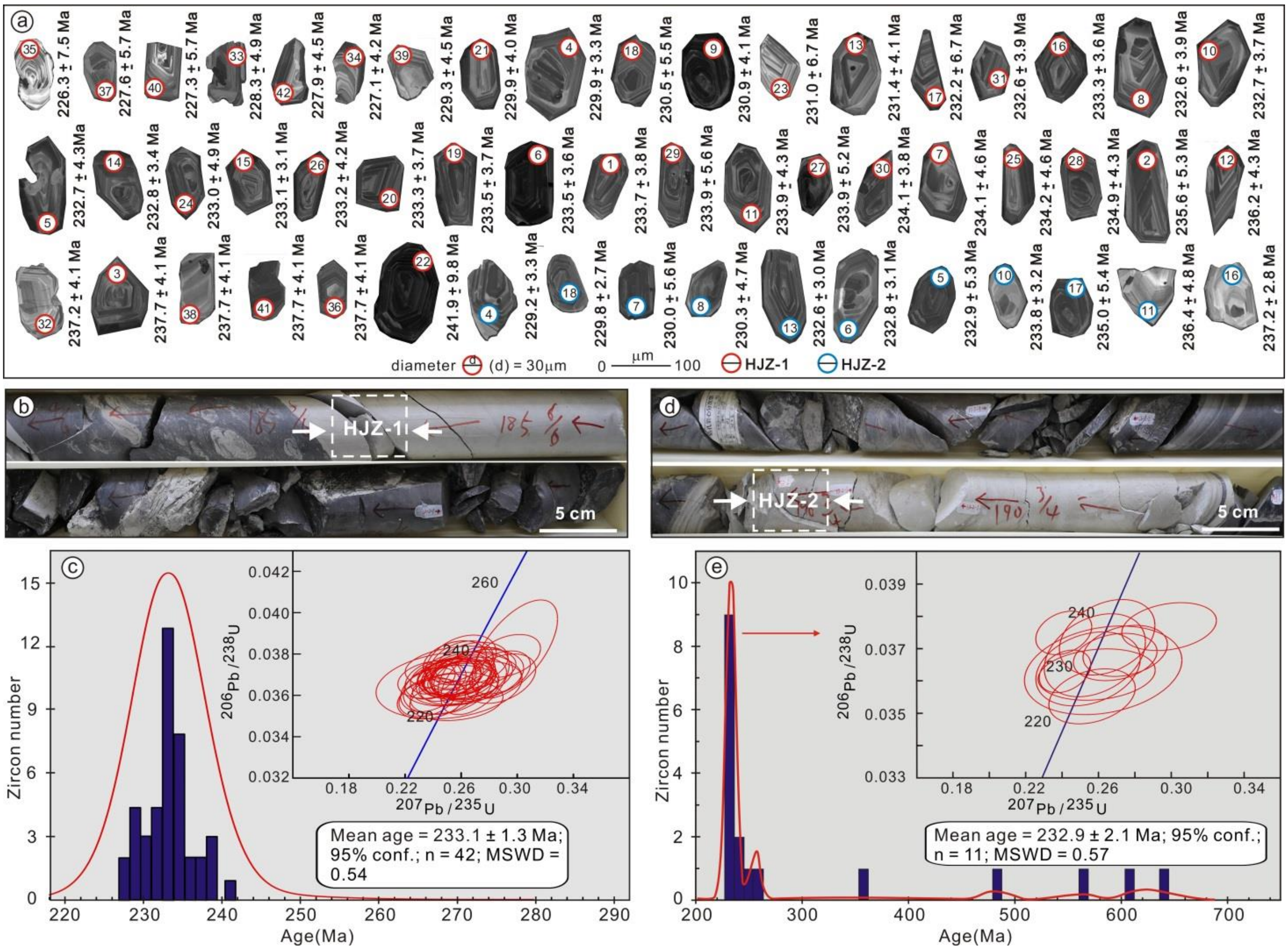

Figure. S2. Representative cathodoluminescence (CL) and tuffaceous claystone images, U-Pb probability density, and U–Pb concordia diagrams for dated zircons from the studied borehole in the Jiyuan Basin. **a**, representative CL images of dated zircons, showing the sites of LA-ICP-MS U–Pb analyses. Red circle = HJZ-1 samples, blue circle = HJZ-2 samples, in each case showing the position of zircon LA-ICP-MS U–Pb age-dating analysis, with numbers within the colored circles showing sample numbers. **b** and **d**, photographs of the ZJ-1 borehole in the Jiyuan Basin showing features of the tuffaceous claystone bed sampled. **c**, U-Pb probability density, and U–Pb concordia diagrams for dated zircons from sample HJZ-1, showing 31 concordant age values distributed in a single peak, with a weighted mean $^{206}Pb/^{238}U$ age of 233.1 ± 1.3 Ma (MSWD = 0.54, n = 42; uncertainties are given at the 2σ level/95% confidence). **e**, U-Pb probability density, and U–Pb concordia diagrams for dated zircons from sample HJZ-2, showing 11 concordant age values distributed in a single peak, with a weighted mean $^{206}Pb/^{238}U$ age of 232.9 ± 2.1 Ma (MSWD = 0.57, n = 11; uncertainties are given at the 2σ level/95% confidence).

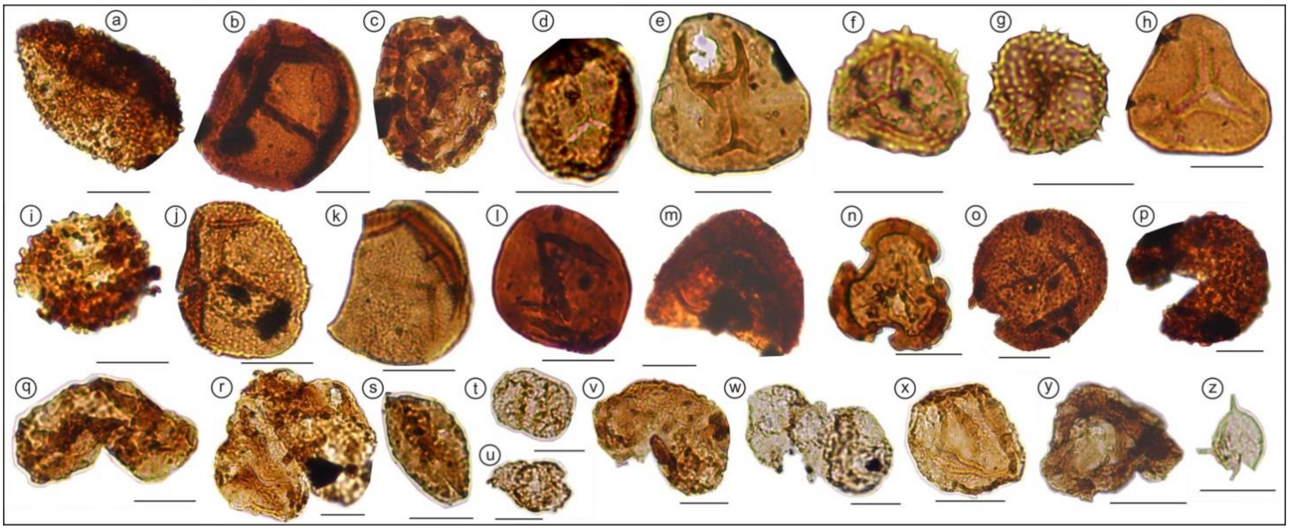

Figure S3. Selected photos of representative palynological genera from studied borehole in the Jiyuan Basin (all scale bars = 20 μm). a = *Baculatisporites* (#JY-8); b = *Crassispora orientalis* (#JY-3); c = *Crassitudisporites* (#JY-3); d = *Cyclogranisporites* (#JY-4); e = *Dictyophyllidites* (#JY-2); f = *Kraeuselisporites* (#JY-3); g = *Lophotriletes* (#JY-11); h = *Matonisporites* (#JY-3); i = *Neoraistrickia* (#JY-8); j, k = *Osmundacidtes* (#JY-3); l = *Punctatisporites* (#JY-3); m = *Torispora* (#JY-3); n = *Triquitrites* (#JY-3); o, p = *Verrucosisporites* (#JY-2); q = *Chordasporites* (#JY-8); r = *Colpectopollis* (#JY-8); s = *Cycadopites* (#JY-11); t = *Caytonipollenites* (#JY-11); u = *Limitisporites* (#JY-9); v = *Piceaepollenites* (#JY-11); w = *Podocarpidites* (#JY-11); x = *Psophosphaera* (#JY-8); y = *Quadraeculina* (#JY-8); z = *Micrhystridium* (#JY-7).

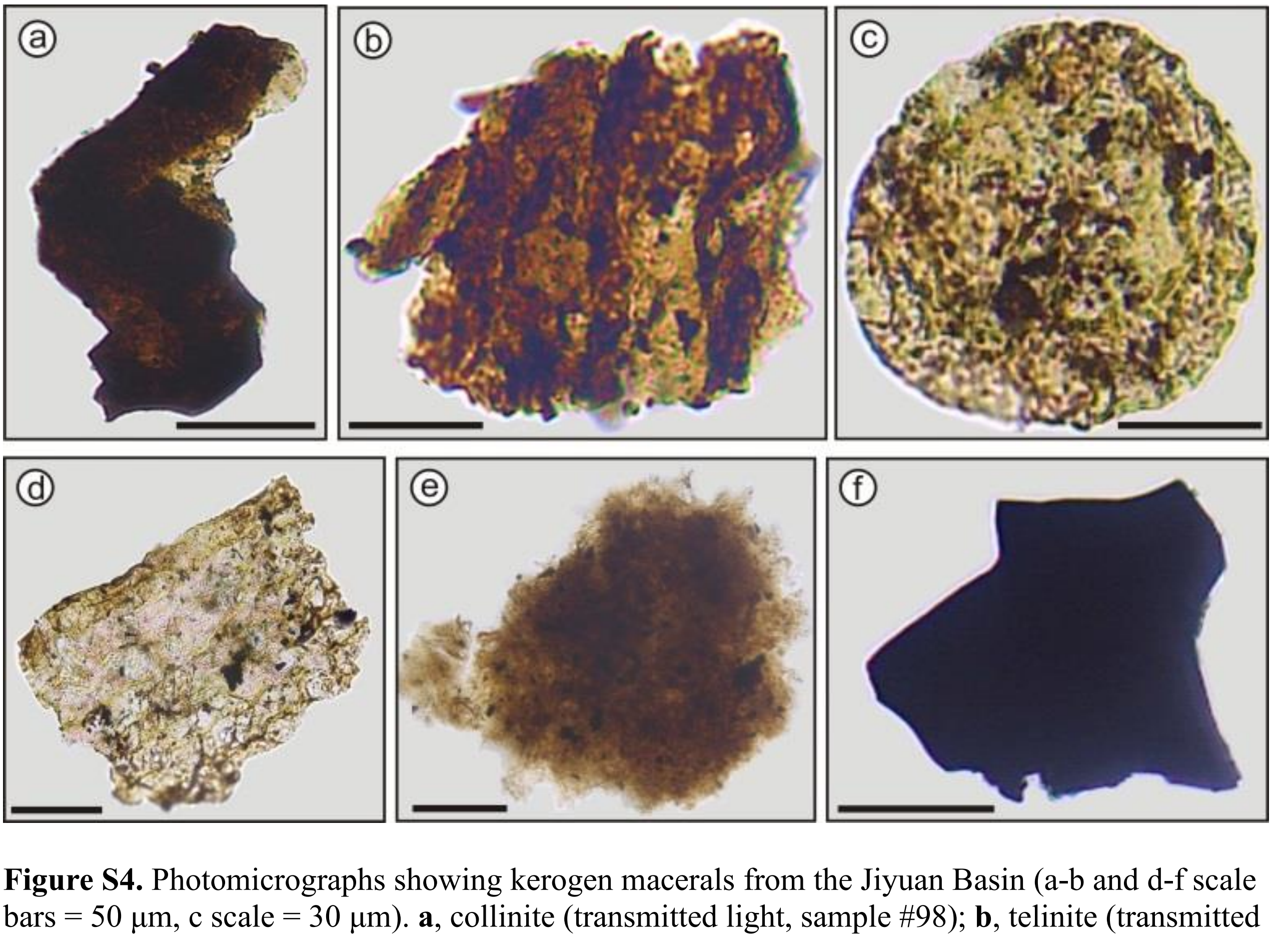

Figure S4. Photomicrographs showing kerogen macerals from the Jiyuan Basin (a-b and d-f scale bars = 50 μm, c scale = 30 μm). **a**, collinite (transmitted light, sample #98); **b**, telinite (transmitted

light, sample #91); **c**, saporopollenite (transmitted light, sample #94); **d**, suberinite (transmitted light, sample #91); **e**, sapropelinite (transmitted light, sample #73); **f**, inertinite (transmitted light, sample #96).

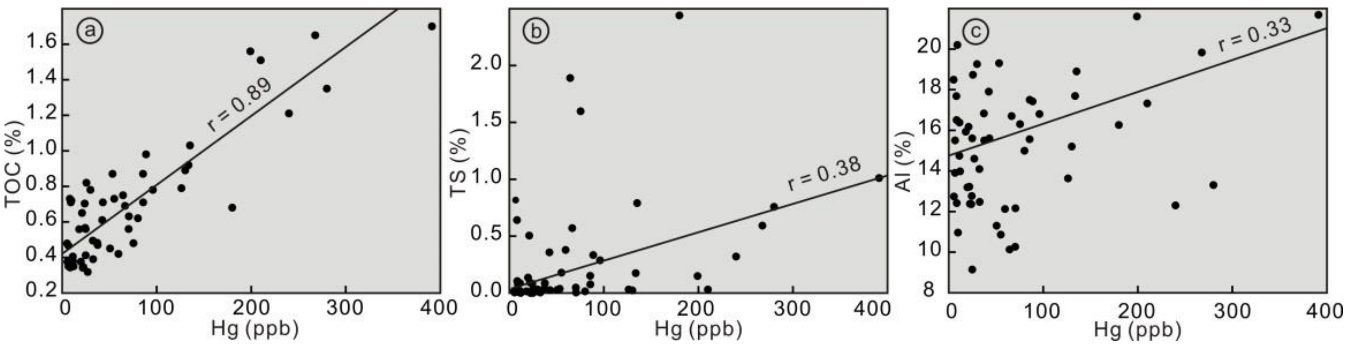

Figure S5. Plots showing mercury (Hg) concentrations versus total organic carbon (TOC), total sulfur (TS), and aluminum (Al) from all samples (n = 55) in the studied borehole. **a**, **b**, and **c**, Plots showing that Hg concentrations show stronger covariation with TOC (r = +0.89) than with TS or Al (r = 0.38 and 0.33 respectively).

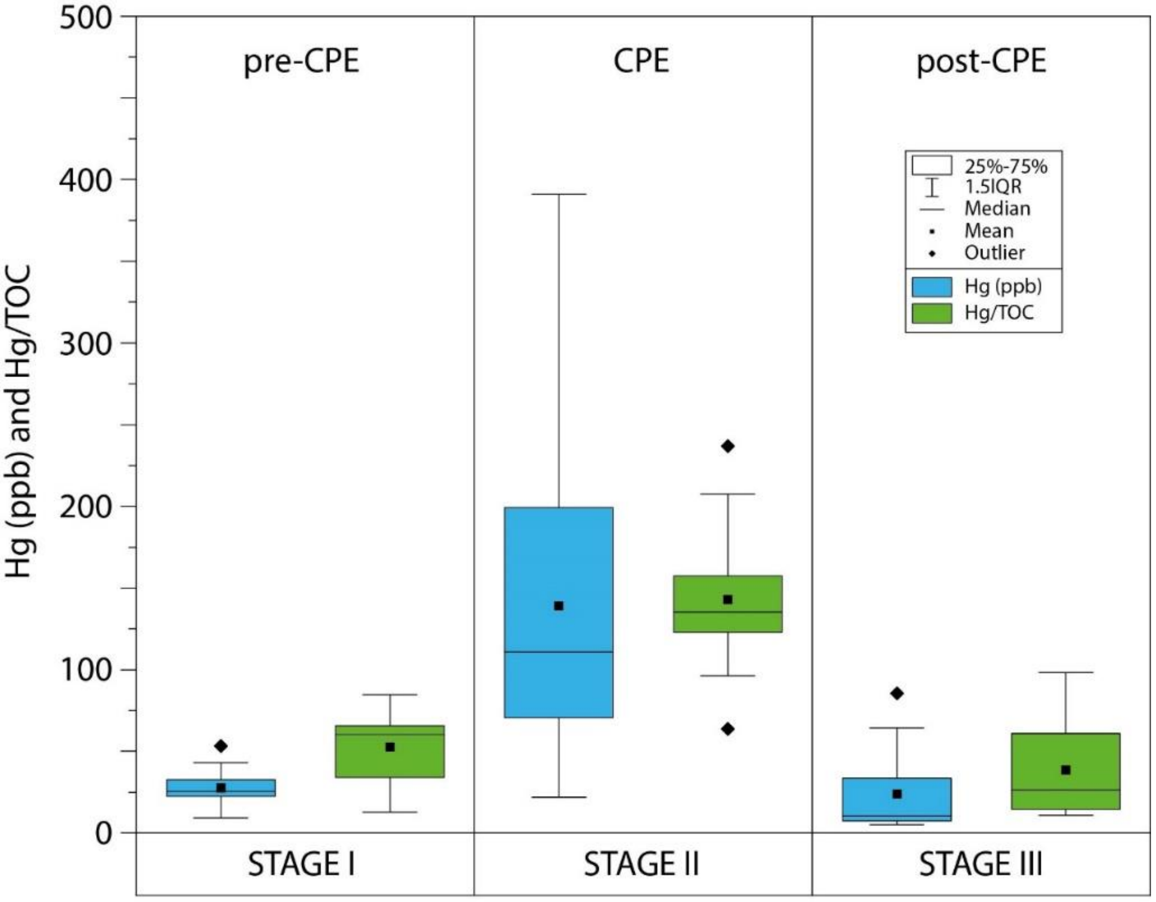

Figure S6. Boxplot of Hg concentrations and Hg/TOC ratios from studied borehole in the Jiyuan Basin.

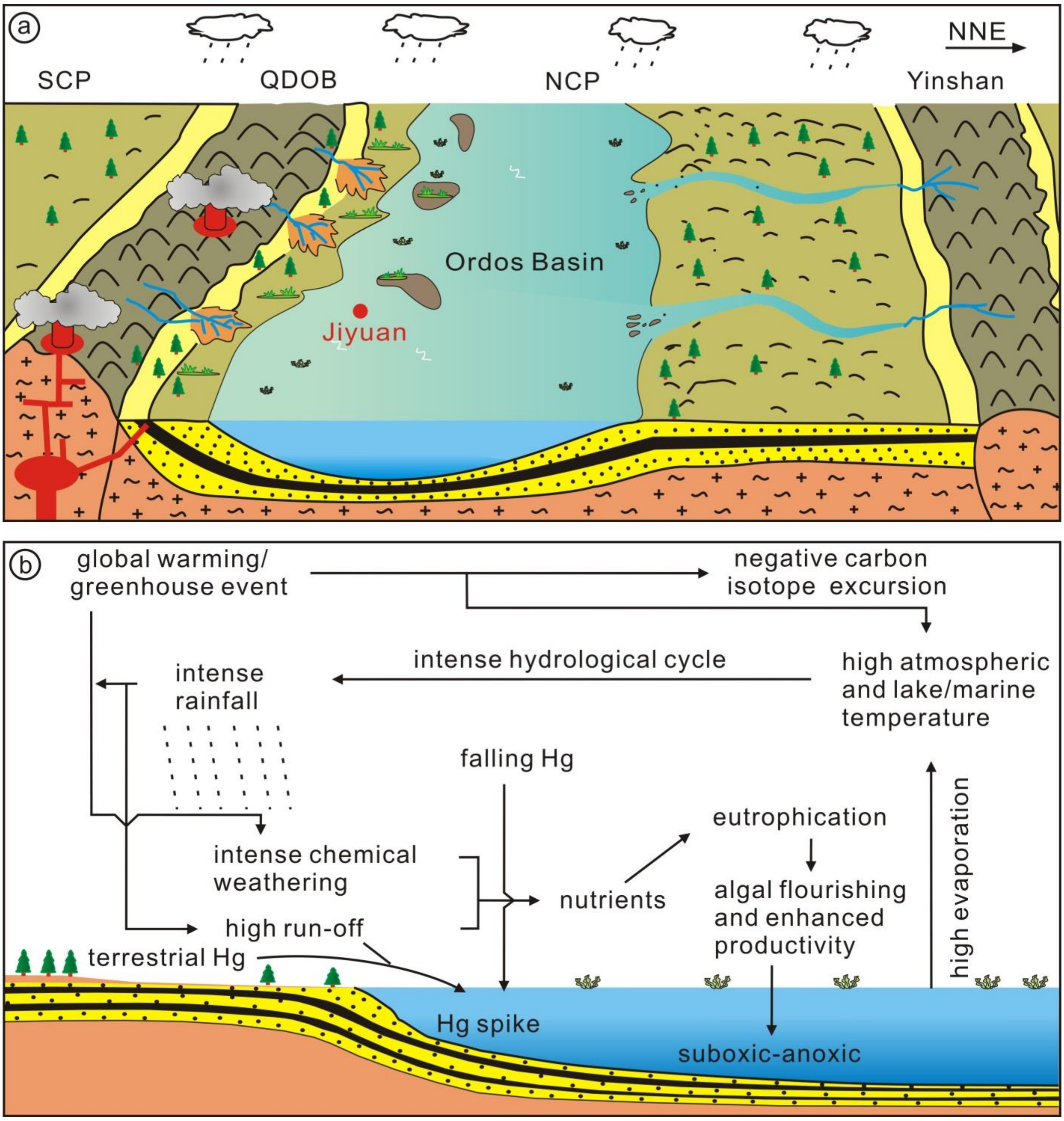

Figure S7. Paleogeographic reconstruction of the NCP and schematic diagram of the terrestrial–lake teleconnections during the CPE interval. **a**, Schematic diagram showing the basin-mountain relationships and the location of the NCP during the CPE interval (modified from reference (21)). **b**, Schematic model linking greenhouse event, enhanced global chemical weathering, and their ecological consequences to the lake realm during the CPE interval. Note: Events are arrayed by cause-and-effect relationships, terrestrial and falling Hg are mainly from global volcanic eruption. Abbreviations: SCP = South China Plate; QDOB = Qinling-Dabie Orogenic Belt; NCP = North China Plate; NNE = North-north-east.

Supplementary Tables

Sample number	Zircon sample number	Content		Th/U	Isotope ratios				rho	Age		Concordance
		Th	U		$^{207}Pb/^{235}U$	$^{207}Pb/^{235}U$	$^{206}Pb/^{238}U$	$^{206}Pb/^{238}U$		$^{206}Pb/^{238}U$	$^{206}Pb/^{238}U$	
		ppm	ppm		Ratio	1sigma	Ratio	1sigma		Age (Ma)	1sigma	
HJZ-1	HJZ-1-1	502.3	850.4	1.23	0.2540	0.0146	0.0369	0.0006	0.2894	233.7	3.8	98%
	HJZ-1-2	500.3	741.9	1.81	0.2598	0.0261	0.0373	0.0007	0.1863	235.6	5.3	99%
	HJZ-1-3	294.3	554.7	1.14	0.2557	0.0149	0.0376	0.0007	0.2998	237.7	4.4	97%
	HJZ-1-4	246.9	503.9	0.80	0.2560	0.0202	0.0363	0.0005	0.1860	229.9	3.3	99%
	HJZ-1-5	324.2	647.9	0.73	0.2497	0.0142	0.0368	0.0007	0.3342	232.7	4.3	97%
	HJZ-1-6	432.3	723.8	0.80	0.2572	0.0187	0.0369	0.0006	0.2171	233.5	3.6	99%
	HJZ-1-7	199.3	433.1	1.46	0.2646	0.0214	0.0370	0.0007	0.2496	234.1	4.6	98%
	HJZ-1-8	249.6	487.2	1.25	0.2402	0.0132	0.0367	0.0006	0.3064	232.6	3.9	93%
	HJZ-1-9	181.8	397.8	1.04	0.2531	0.0221	0.0365	0.0007	0.2048	230.9	4.1	99%
	HJZ-1-10	251.6	428.9	0.93	0.2394	0.0161	0.0368	0.0006	0.2424	232.7	3.7	93%
	HJZ-1-11	323.5	628.9	0.59	0.2604	0.0139	0.0369	0.0007	0.3486	233.9	4.3	99%
	HJZ-1-12	325.4	493.2	1.28	0.2604	0.0148	0.0376	0.0007	0.3299	236.2	4.3	98%
	HJZ-1-13	275.9	465.9	1.48	0.2587	0.0188	0.0365	0.0007	0.2504	231.4	4.1	99%
	HJZ-1-14	324.0	612.8	0.95	0.2554	0.0125	0.0368	0.0005	0.3029	232.8	3.4	99%
	HJZ-1-15	506.1	698.0	1.08	0.2400	0.0105	0.0368	0.0005	0.3100	233.1	3.1	93%
	HJZ-1-16	329.4	584.4	1.18	0.2541	0.0134	0.0369	0.0006	0.3007	233.3	3.6	98%
	HJZ-1-17	394.6	713.8	0.87	0.2701	0.0161	0.0367	0.0011	0.4912	232.2	6.7	95%
	HJZ-1-18	181.7	383.8	1.49	0.2507	0.0210	0.0364	0.0009	0.2891	230.5	5.5	98%
	HJZ-1-19	444.9	750.8	0.73	0.2517	0.0123	0.0369	0.0006	0.3319	233.5	3.7	97%
	HJZ-1-20	312.7	469.5	0.82	0.2609	0.0149	0.0368	0.0006	0.2831	233.3	3.7	99%
	HJZ-1-21	400.2	749.6	0.73	0.2390	0.0161	0.0358	0.0006	0.2672	229.9	4.0	95%
	HJZ-1-22	454.4	629.5	1.10	0.2985	0.0201	0.0382	0.0016	0.6107	241.9	9.8	90%
	HJZ-1-23	170.4	235.1	0.82	0.2516	0.0344	0.0365	0.0011	0.2158	231.0	6.7	98%
	HJZ-1-24	354.0	627.8	1.49	0.2885	0.0164	0.0368	0.0008	0.3781	233.0	4.9	90%
	HJZ-1-25	275.1	479.0	0.89	0.2570	0.0181	0.0370	0.0007	0.2812	234.2	4.6	99%
	HJZ-1-26	341.6	618.1	0.79	0.2667	0.0171	0.0368	0.0007	0.2848	233.2	4.2	97%
	HJZ-1-27	336.3	640.3	0.64	0.2715	0.0220	0.0370	0.0008	0.2780	233.9	5.2	95%
	HJZ-1-28	346.2	622.9	0.97	0.2878	0.0144	0.0371	0.0007	0.3722	234.9	4.3	91%
	HJZ-1-29	277.0	564.0	0.92	0.2637	0.0131	0.0369	0.0009	0.4928	233.9	5.6	98%
	HJZ-1-30	435.3	658.8	0.95	0.2599	0.0147	0.0370	0.0006	0.2941	234.1	3.8	99%

Sample number	Zircon sample number	Content		Th/U	Isotope ratios				rho	Age		Concordance
		Th	U		$^{207}Pb/^{235}U$	$^{207}Pb/^{235}U$	$^{206}Pb/^{238}U$	$^{206}Pb/^{238}U$		$^{206}Pb/^{238}U$	$^{206}Pb/^{238}U$	
		ppm	ppm		Ratio	1sigma	Ratio	1sigma		Age (Ma)	1sigma	
HJZ-1	HJZ-1-31	219.8	473.0	0.93	0.2594	0.0168	0.0367	0.0006	0.2621	232.6	3.9	99%
	HJZ-1-32	270.4	387.2	0.70	0.2678	0.0171	0.0375	0.0007	0.2864	237.2	4.1	93%
	HJZ-1-33	209.6	428.6	0.49	0.2557	0.0149	0.0376	0.0007	0.2998	228.3	4.9	95%
	HJZ-1-34	335.8	458.7	0.73	0.2390	0.0161	0.0358	0.0006	0.2672	227.1	4.2	91%
	HJZ-1-35	312.0	444.0	0.70	0.2568	0.0220	0.0357	0.0006	0.2060	226.3	7.5	97%
	HJZ-1-36	265.8	362.4	0.73	0.2939	0.0151	0.0379	0.0007	0.3513	239.9	3.5	90%
	HJZ-1-37	249.0	486.4	0.51	0.2365	0.0136	0.0359	0.0006	0.3049	227.6	5.7	96%
	HJZ-1-38	231.0	359.8	0.64	0.2601	0.0166	0.0376	0.0009	0.3571	238.5	3.8	92%
	HJZ-1-39	284.1	480.8	0.59	0.2383	0.0140	0.0363	0.0007	0.3305	229.3	4.5	98%
	HJZ-1-40	287.1	429.0	0.67	0.2668	0.0240	0.0367	0.0006	0.2160	227.3	5.7	93%
	HJZ-1-41	280.7	456.3	0.62	0.2557	0.0149	0.0376	0.0007	0.2998	239.2	4.3	90%
	HJZ-1-42	429.8	595.8	0.72	0.2483	0.0160	0.0361	0.0006	0.2749	227.9	4.5	91%
	Plešovice-1	99.8	907.1	0.11	0.4316	0.0178	0.0546	0.0007	0.3316	342.8	4.6	93%
	Plešovice-2	108.0	952.5	0.11	0.4438	0.0175	0.0545	0.0007	0.3426	342.4	4.5	91%
	Plešovice-3	102.3	924.1	0.11	0.3749	0.0143	0.0538	0.0012	0.5630	336.7	4.7	90%
	Plešovice-4	101.5	908.2	0.11	0.3690	0.0221	0.0541	0.0008	0.2321	339.9	4.6	93%
	91500-1	24.6	71.1	0.35	2.1240	0.2841	0.1794	0.0042	0.1742	1063.5	22.9	91%
	91500-2	27.3	76.1	0.36	1.5764	0.1288	0.1790	0.0035	0.2406	1061.4	19.3	90%
	91500-3	26.1	76.2	0.34	1.9433	0.1055	0.1789	0.0034	0.3535	1060.7	18.8	96%
	91500-4	26.0	75.1	0.35	1.7571	0.1036	0.1795	0.0036	0.3358	1064.1	19.4	96%
	91500-5	26.0	76.1	0.34	1.9031	0.1158	0.1795	0.0036	0.3265	1064.5	19.5	98%
	91500-6	25.1	73.3	0.34	1.7973	0.1106	0.1788	0.0035	0.3194	1060.4	19.2	98%
	91500-7	24.6	74.1	0.33	1.8627	0.1515	0.1781	0.0039	0.2674	1056.6	21.2	98%
	91500-8	24.8	73.0	0.34	1.8377	0.1125	0.1802	0.0034	0.3060	1068.3	18.5	99%
	91500-9	26.8	76.6	0.35	2.1470	0.1265	0.1794	0.0039	0.3660	1064.0	21.2	91%
	91500-10	26.2	75.9	0.34	1.5534	0.1186	0.1789	0.0042	0.3076	1060.9	22.4	90%
	91500-11	26.7	77.0	0.35	1.8224	0.0929	0.1791	0.0034	0.3694	1062.1	18.5	99%
	91500-12	27.1	76.3	0.35	1.8780	0.1025	0.1792	0.0031	0.3150	1062.8	16.9	99%
	91500-13	26.4	69.5	0.38	1.7751	0.1015	0.1801	0.0029	0.2784	1067.8	15.7	97%
	91500-14	26.4	70.0	0.38	1.9253	0.0860	0.1782	0.0031	0.3934	1057.1	17.2	96%
	91500-15	24.5	72.5	0.34	1.8165	0.1048	0.1791	0.0041	0.3933	1062.1	22.2	98%
	91500-16	25.1	72.4	0.35	1.8839	0.1073	0.1792	0.0032	0.3140	1062.8	17.5	98%
	91500-17	23.9	68.2	0.35	1.8221	0.0983	0.1792	0.0029	0.2976	1062.7	15.7	99%

Sample number	Zircon sample number	Content		Th/U	Isotope ratios				rho	Age		Concordance
		Th	U		$^{207}Pb/^{235}U$	$^{207}Pb/^{235}U$	$^{206}Pb/^{238}U$	$^{206}Pb/^{238}U$		$^{206}Pb/^{238}U$	$^{206}Pb/^{238}U$	
		ppm	ppm		Ratio	1sigma	Ratio	1sigma		Age (Ma)	1sigma	
HJZ-1	91500-18	24.0	72.4	0.33	1.8375	0.1604	0.1787	0.0030	0.1919	1060.0	16.4	99%
	91500-19	25.2	76.0	0.33	1.8629	0.1096	0.1796	0.0030	0.2880	1064.9	16.6	99%
	91500-20	24.2	73.3	0.33	1.7291	0.1045	0.1789	0.0036	0.3284	1060.7	19.4	96%
HJZ-2	HJZ-2-1	338.8	465.2	0.73	0.2970	0.0183	0.0401	0.0008	0.3402	253.6	5.2	95%
	HJZ-2-2	309.8	174.8	1.77	0.8704	0.1930	0.0980	0.0276	1.2685	602.4	22.8	94%
	HJZ-2-3	345.9	617.1	0.56	0.2784	0.0168	0.0408	0.0007	0.2680	258.0	4.1	96%
	HJZ-2-4	245.5	503.2	0.49	0.2590	0.0212	0.0362	0.0005	0.1766	229.2	3.3	98%
	HJZ-2-5	860.7	604.5	1.42	0.2580	0.0183	0.0368	0.0008	0.3240	232.9	5.3	99%
	HJZ-2-6	1102.6	948.5	1.16	0.2988	0.0172	0.0377	0.0005	0.2533	232.8	3.1	96%
	HJZ-2-7	1071.4	711.3	1.51	0.2626	0.0286	0.0363	0.0009	0.2290	230.0	5.6	97%
	HJZ-2-8	257.1	495.9	0.52	0.2543	0.0189	0.0364	0.0008	0.2788	230.4	4.7	99%
	HJZ-2-9	297.8	269.0	1.11	0.4121	0.2171	0.0565	0.0082	0.2751	354.5	5.0	98%
	HJZ-2-10	1625.9	682.7	2.38	0.2747	0.0153	0.0369	0.0005	0.2537	233.8	3.3	94%
	HJZ-2-11	1516.6	681.8	2.22	0.2648	0.0173	0.0374	0.0008	0.3157	236.4	4.8	99%
	HJZ-2-12	60.3	93.0	0.65	0.5812	0.0443	0.0777	0.0023	0.3924	482.1	13.9	96%
	HJZ-2-13	1102.6	948.5	1.16	0.2492	0.0132	0.0367	0.0005	0.3133	232.7	3.0	96%
	HJZ-2-14	551.1	300.1	1.84	0.7639	0.0739	0.0911	0.0037	0.4229	562.3	22.0	97%
	HJZ-2-15	377.4	256.8	1.47	0.9144	0.0345	0.1045	0.0031	0.7961	640.6	18.3	97%
	HJZ-2-16	2323.5	1037.0	2.24	0.2409	0.0101	0.0375	0.0004	0.2813	237.2	2.8	92%
	HJZ-2-17	1074.5	721.4	1.49	0.2565	0.0157	0.0355	0.0006	0.3025	235.0	5.4	91%
	HJZ-2-18	2591.9	1086.7	2.39	0.2416	0.0093	0.0363	0.0004	0.3102	230.8	2.7	95%
	Plešovice-1	107.7	788.6	0.14	0.4041	0.0142	0.0534	0.0007	0.3482	335.6	4.0	97%
	Plešovice-2	97.9	742.3	0.13	0.4128	0.0153	0.0534	0.0006	0.3246	335.3	3.9	95%
	Plešovice-3	97.9	742.3	0.13	0.4128	0.0153	0.0534	0.0006	0.3246	335.3	3.9	95%
	Plešovice-4	108.2	829.8	0.17	0.3749	0.0143	0.0538	0.0012	0.5630	337.6	7.1	95%
	91500-1	24.3	72.0	0.34	1.5728	0.0836	0.1789	0.0027	0.2834	1060.8	14.7	90%
	91500-2	25.9	76.7	0.34	1.5790	0.1011	0.1788	0.0031	0.2669	1060.3	16.7	90%
	91500-3	24.9	69.7	0.36	2.1276	0.0979	0.1795	0.0025	0.3029	1064.1	13.7	91%
	91500-4	25.3	78.7	0.32	2.1214	0.1735	0.1796	0.0031	0.2134	1064.5	17.1	91%
	91500-5	24.5	72.6	0.34	1.6449	0.0915	0.1795	0.0033	0.3259	1064.5	17.8	92%
	91500-6	24.9	75.6	0.33	1.6301	0.1081	0.1790	0.0030	0.2567	1061.3	16.7	92%
	91500-7	26.0	74.8	0.35	2.0555	0.0960	0.1788	0.0034	0.4056	1060.4	18.5	93%
	91500-8	24.3	69.5	0.35	2.0703	0.1495	0.1794	0.0032	0.2446	1063.6	17.3	93%

Sample number	Zircon sample number	Content		Th/U	Isotope ratios				rho	Age		Concordance
		Th	U		$^{207}Pb/^{235}U$	$^{207}Pb/^{235}U$	$^{206}Pb/^{238}U$	$^{206}Pb/^{238}U$		$^{206}Pb/^{238}U$	$^{206}Pb/^{238}U$	
		ppm	ppm		Ratio	1sigma	Ratio	1sigma		Age (Ma)	1sigma	
HJZ-2	91500-9	25.7	71.3	0.36	1.6851	0.1269	0.1793	0.0035	0.2557	1063.4	18.9	94%
	91500-10	23.4	72.2	0.32	2.0153	0.1282	0.1790	0.0035	0.3047	1061.5	19.0	94%

Table S1. Results of two tuffaceous claystone samples for zircon U–Pb dating from the studied borehole in the Jiyuan basin of the NCP.

Algae and spore-pollen	Tanzhuang Formation						Anyao Formation				
	JY-11	JY-10	JY-9	JY-8	JY-7	JY-6	JY-5	JY-4	JY-3	JY-2	JY-1
Leiosphaeridia		6	6	16		15	24	10	2	12	6
Granodiscus	4	2	4	5		60	210	12	1	5	5
Verrucosphaera tuberculata	1	2	1	30		15	35	21		28	5
Operculodinium multispinosum								1			
Micrhystridium					500						
**Sum of algae**	5	10	11	51	500	90	269	44	3	45	16
Neoraistrickia (h)	1							1	1		
Baculatisporites (h)	3	2		1	3	3	2	1	3	1	3
Osmundacidites (h)	6	5	10	32	10	9	8	5	6	3	5
Cadargasporites (h)	2							1			
Densosporites (h)	1	1	2							2	1
Crassispora (h)	2	2	3	2	3	2	2	3		1	7
Crassispora orientalis (h)		1	2	3	5	4				1	
Kraeuselisporites (h)		1	1	3	5	4				5	3
Kraeuselisporites disparillis (h)			2	1	3	2					
Cibotiumspora (h)	1		3	1	2	3		2			
Triquitrites (h)	5		1	4	6	5		3		3	3
Triquitrites sagmarius (h)			2	1	3	3					
Asseretospora (h)		1							1		
Crassitudisporites (h)				2	2	2	1	2	2	1	4
Lophotriletes (h)	3	2	1	2	3	2	1	3	3	8	7
Anapiculatisporites (h)			2	2	4	5	1	3	6	1	5
Verrucosisporites (h)	5	8	2	8	3	2	1	1	4	10	3
Matonisporites (h)			1						1	2	
Dictyophyllidites (h)		1				1	1	1			
Toroisporis (h)						2	1				
Cyathidites (h)	2	1	2	1	3	2	1	2	1	2	
Cyathidites minor (h)			2	3	2						
Laevigatosporites (h)	1	1	2	2	3	3	1	2		6	
Laevigatosporites major (h)			2	1	2					3	
Torispora securis (h)	1			1	2	2					
Punctatosporites (h)										1	
Aratrisporites (h)									1		
Calamospora (h)			2	1			4			2	
Punctatisporites (h)	3	4	1	7	1	3	5	4	3	7	1
Punctatisporites minutus (h)		1	1	7	5	3	3	2		6	
Punctatisporites giganteus (h)		5	4	4	3	5	7		1	3	1
Cyclogranisporites (h)	24	14	6	29	10	19	15	5	2	8	1
Leiotriletes (h)	1		1	2						1	
**Sum of spores**	61	50	55	120	83	86	54	41	35	77	44
Pinaceae (X)		1								1	
Piceaepollenites (X)		1					1				
Pinuspollenites (X)		1	2	1	2	1					
Podocarpidites (X)	1		1	1	3	2		5	1		1
Cycadopites (h)		3	2	1	2	2	4	1	3	3	1
Chasmatosporites (X)		4	1	3	5	4	2	5	2	4	3
Psophosphaera (X)	12	4	1	1	1	2	1	1	4	3	3
Psophosphaera sp. (X)							3	3		1	2
Inaperturopollenites (X)			3	7	3	2	5	5		4	3

Algae and spore-pollen	Tanzhuang Formation						Anyao Formation				
	JY-11	JY-10	JY-9	JY-8	JY-7	JY-6	JY-5	JY-4	JY-3	JY-2	JY-1
Concentrisporites (X)										2	
Perinopollenites (X)		1									
Chordasporites (X)	1								3	1	5
Colpectopollis (X)									2		
Vesicaspora (X)	11	7	5	3	3	2	5	4	4	3	3
Florinites (X)				2	2						
Caytonipollenites (X)		1	2		1						
Cordaitina (X)	4	2	1		1	2	2	3	2	1	3
Alisporites (h)							1				
Limitisporites (X)								1			
Taeniaesporites (X)									1		
Callialasporites (X)		2					1	2	2	2	3
Parvisaccites (X)										2	
Quadraeculina (X)	2	1					1		2		
Cerebropollenites (X)	1		2					2		1	
Rotundipollis (X)	17	13	2	4			4	7	3	8	7
Pseudopicea (X)	22	7	2	1	2	1	10	5	19	7	6
Protopinus (X)	6	8	7				3	5	2	3	3
Protoconiferus (X)	4	2	5				2	5	8	9	9
Paleoconiferus (X)	23	14	10	2			8	9	12	3	10
**Sum of pollen**	104	72	46	26	25	18	53	63	70	58	62
**Sum of Spore-pollen**	165	122	101	146	108	104	107	104	105	135	106
**Hygrophytic (h)**	61	53	57	121	85	88	59	42	38	82	45
**Xerophytic (x)**	104	69	44	25	23	16	48	62	67	55	61

Table S2. Results of the quantitative algae and spore-pollen analysis (number).

**Botanical affinity**	**Sporomorph genera**	**Botanical affinity**	**Sporomorph genera**
Horsetails	*Calamospora*	Conifers (Pinaceae)	*Pinuspollenites*
Ferns (Dipteridaceae/Matoniaceae)	*Dictyophyllidites*		*Piceaepollenites*
	Matonisporites	Conifers (Podocarpaceae)	*Podocarpidites*
Ferns (Dipteridaceae)	*Verrucosisporites*		*Taeniaesporites*
Ferns (Osmundaceae)	*Baculatisporites*		*Quadraeculina*
	Osmundacidites	Conifers	*Pinaceae*
Ferns (Pteridaceae)	*Asseretospora*		*Pseudopicea*
Ferns (Marattiaceae)	*Toroisporis*		*Protopinus*
‘filicalean’ ferns	*Laevigatosporites*		*Protoconiferus*
	Torispora		*Paleoconiferus*
	Punctatosporites		*Psophosphaera*
	Punctatisporites		*Vesicaspora*
	Cyclogranisporites		*Florinites*
	Leiotriletes	Gymnosperms	*Chordasporites*
	Lophotriletes		*Cordaitina*
	Anapiculatisporites		*Colpectopollis*
	Triquitrites		*Caytonipollenites*
	Crassitudisporites		*Parvisaccites*
Ferns (Dicksoniaceae/Cyatheaceae)	*Cibotiumspora*		*Rotundipollis*
	Cyathidites	Mosses	*Cadargasporites*
Cycadophytes	*Cycadopites*	Lycopsids	*Aratrisporites*
	Concentrisporites		*Neoraistrickia*
Cycads	*Chasmatosporites*		*Densosporites*
Conifers (Taxodiaceae)	*Inaperturopollenites*		*Kraeuselisporites*
	Perinopollenites		*Crassispora*
	Callialasporites	Seed ferns	*Alisporites*
	Cerebropollenites		*Limitisporites*

Table S3. Botanical affinity for dispersed Upper Triassic miospores from the studied strata. Note: This summary is mainly based upon published references on the botanical affinity of dispersed spores and ecology of Mesozoic plants: (15–29).

Formation	Sample	Vitrinite	Exinite	Sapropelinite	Inertinite	T_{max}/°C
Anyao	53	30.1	32.8	27.3	9.8	442
	54					
	55	35.6	30.9	26.3	7.2	
	56					
	57	28.1	31.2	26.5	14.2	
	58	34.1	36.0	24.7	5.2	448
	59					
	60	34.6	27.2	29.5	8.7	
	61					
	62	31.6	30.0	28.2	10.2	445
	63					
	64	24.5	21.8	40.5	13.2	
	65					
	66	26.0	26.5	37.8	9.7	
	67					
	68	24.1	41.2	21.5	13.2	444
	69					
	70	22.6	22.1	40.2	15.1	
	71	19.3	21.4	38.1	21.2	445
	72					
	73	19.0	24.8	37.3	18.9	
	74					
Tanzhuang	75	17.9	29.3	39.6	13.2	454
	76					
	77	27.6	22.3	41.2	8.9	
	78					
	79	13.4	30.5	37.3	18.8	
	80					
	81	17.6	18.9	43.2	20.3	441
	82					
	83	18.5	20.3	39.9	21.3	
	84					
	85	16.2	23.4	40.9	19.5	
	86					
	87	17.6	24.6	38.9	18.9	443
	88					
	89	28.8	22.0	32.5	16.7	
	90					
	91	27.9	20.8	38.1	13.2	
	92	31.3	42.4	17.6	8.7	445
Tanzhuang	93					
	94	55.1	37.5	2.2	5.2	
	95					
	96	45.2	41.7	1.9	11.2	
	97					
	98	45.4	36.5	7.9	10.2	
	99					
	100	53.6	35.1	3.5	7.8	447
	101					
	102	47.4	38.1	1.9	12.6	
	103					
	104	43.8	39.7	3.4	13.1	
	105					

Formation	Sample	Vitrinite	Exinite	Sapropelinite	Inertinite	T_{max}/°C
Tanzhuang	106	40.7	31.8	8.2	19.3	
	107	42.5	37.9	3.3	16.3	

Table S4. Results of Kerogen macerals (%) including vitrinite, exinite, sapropelinite, and inertinite and the peak temperature of rock pyrolysis (T_{max}, °C) from the studied borehole in the Jiyuan basin of the NCP.

Organic matter		Diagenetic stage division	
maturity	T_{max}/°C		
immaturate	< 430	early diagenetic stage	A
semi-mature	430 - 435		B
low-mature to mature	435 - 460	middle diagenetic stage	A
high-mature	460 - 490		B
post-mature	> 490	late diagenetic and metamorphic stages	

Table S5. Relationship between maturity of organic matter and diagenetic stage according to the China national standard (SY/T 5477-2003).

Formation	Sample	$\delta^{13}C_{org}$	TOC	Hg	Hg/TOC	P	Al	P/Al	Th	U	Th/U	TS	%C	%N	C/N
	53	-23.2	0.35	11.97	34.20	0.125	13.98	89.41	12.6	2.59	4.86	0.00	27.15	1.17	23.21
	54	-23.1	0.39	32.67	83.77	0.143	12.47	114.68	12.7	2.51	5.06	0.00	26.96	1.13	23.86
	55	-23.1	0.38	11.42	30.05	0.133	16.38	81.20	15.0	2.95	5.08	0.02	26.52	1.12	23.68
	56	-23.4	0.35	22.37	63.91	0.145	12.38	117.12	13.0	2.42	5.37	0.00	25.22	1.12	22.52
	57	-22.8	0.70	23.87	34.10	0.118	12.36	95.47	11.2	2.35	4.77	0.00	30.43	1.43	21.28
	58	-24.1	0.82	25.53	31.13	0.133	18.73	71.01	17.5	3.44	5.09	0.02	23.12	1.12	20.64
	59	-24.0	0.87	53.21	61.16	0.132	19.30	68.39	17.9	3.79	4.72	0.04	22.83	1.02	22.38
	60	-24.3	0.78	29.85	38.27	0.153	19.26	79.44	18.4	3.64	5.05	0.03	31.03	1.41	22.01
	61	-22.9	0.48	37.09	77.27	0.133	16.83	79.03	14.1	3.01	4.68	0.04	25.67	1.15	22.32
	62	-25.0	0.71	9.04	12.73	0.136	20.20	67.33	17.1	3.25	5.26	0.08	30.87	1.32	23.39
Anyao	63	-23.4	0.47	37.50	79.79	0.145	15.50	93.55	14.1	2.78	5.07	0.09	25.79	1.13	22.82
	64	-23.7	0.32	26.93	84.16	0.154	14.60	105.48	13.0	2.55	5.10	0.01	31.23	1.35	23.13
	65	-23.0	0.49	32.47	66.27	0.130	14.09	92.26	14.2	2.68	5.30	0.01	31.30	1.39	22.50
	66	-23.5	0.41	24.85	60.61	0.101	9.14	110.50	9.9	2.01	4.94	0.00	27.83	1.25	22.26
	67	-25.1	0.56	24.79	44.27	0.139	15.60	89.10	17.1	3.40	5.03	0.03	30.06	1.43	21.02
	68	-23.4	0.71	43.01	60.58	0.142	15.60	91.03	15.5	3.52	4.40	0.03	32.13	1.53	21.00
	69	-24.5	0.56	17.99	32.13	0.145	15.93	91.02	15.2	3.59	4.23	0.01	29.89	1.56	19.16
	70	-23.0	0.63	70.53	111.95	0.141	12.16	115.95	12.0	2.39	5.02	0.00	34.56	1.83	18.89
	71	-26.5	0.71	85.60	120.56	0.237	17.50	135.26	15.0	3.57	4.20	0.08	33.87	1.93	17.55
	72	-28.3	1.56	199.08	127.62	0.401	21.60	185.65	16.8	6.85	2.45	0.15	31.69	1.99	15.92
	73	-29.8	1.35	279.99	207.40	0.423	13.30	318.05	25.9	19.70	1.31	0.76	33.23	1.92	17.31
	74	-28.5	1.51	209.99	139.07	0.580	17.32	334.87	28.8	20.20	1.43	0.03	34.31	2.09	16.42
	75	-27.6	0.56	70.23	125.41	0.286	10.26	278.75	10.9	3.61	3.02	0.05	32.15	1.93	16.66
	76	-27.9	0.45	50.57	112.38	0.122	11.30	107.96	8.9	4.50	1.98	0.03	32.13	1.91	16.82
	77	-28.7	0.79	126.11	159.63	0.212	13.63	155.54	12.1	5.70	2.12	0.03	34.35	1.89	18.17
	78	-30.3	1.21	239.75	198.14	0.288	12.30	234.15	13.1	8.60	1.52	0.32	33.56	2.03	16.53
	79	-27.8	0.89	130.12	146.20	0.189	15.20	124.34	18.6	8.78	2.12	0.02	35.12	1.95	18.01
	80	-26.5	0.62	80.12	129.23	0.139	14.99	92.73	33.1	9.23	3.59	0.01	32.13	1.93	16.65
	81	-29.3	0.69	66.42	96.26	0.298	16.70	178.44	27.2	13.20	2.06	0.57	33.23	1.98	16.78
	82	-30.5	1.03	135.23	131.29	0.308	18.90	162.96	25.2	17.10	1.47	0.79	32.53	2.06	15.79
	83	-32.7	1.70	267.58	157.40	0.462	21.68	213.10	29.1	23.50	1.24	1.01	33.21	2.06	16.12
	84	-29.5	1.65	391.00	236.97	0.195	19.83	98.34	18.9	12.70	1.49	0.59	34.67	1.93	17.96
	85	-27.4	0.92	133.69	145.32	0.131	17.69	74.05	16.6	4.16	3.99	0.17	33.76	1.97	17.14
	86	-24.9	0.42	59.50	141.67	0.141	12.12	116.34	13.5	3.58	3.77	0.38	32.13	1.93	16.65
	87	-25.7	0.48	75.35	156.98	0.139	16.30	85.28	15.2	5.05	3.01	1.60	30.87	1.81	17.06
	88	-27.2	0.68	88.73	130.49	0.365	16.26	224.48	11.2	6.99	1.60	2.44	32.23	1.72	18.74
	89	-25.7	0.98	179.85	183.52	0.231	17.42	132.61	12.7	6.50	1.95	0.33	36.81	1.85	19.90
	90	-24.8	0.78	95.89	122.94	0.151	16.80	89.88	21.9	7.30	3.00	0.29	33.91	1.73	19.60
Tanzhuang	91	-23.8	0.34	21.73	63.91	0.127	15.56	81.62	16.1	3.02	5.33	0.15	30.13	1.38	21.83
	92	-23.9	0.87	85.50	98.28	0.126	13.22	95.31	12.8	2.58	4.96	0.09	28.79	1.23	23.41
	93	-24.7	0.75	64.26	85.68	0.108	10.13	106.61	12.0	3.04	3.95	1.89	27.56	1.12	24.61
	94	-24.2	0.73	8.13	11.14	0.181	17.68	102.38	25.6	7.83	3.27	0.64	31.74	1.23	25.80
	95	-24.3	0.61	42.30	69.34	0.163	17.90	91.06	17.8	5.65	3.15	0.36	26.52	1.03	25.75
	96	-24.0	0.48	5.17	10.77	0.152	18.49	82.21	18.2	4.51	4.04	0.02	23.87	0.91	26.23
	97	-24.0	0.47	6.66	14.17	0.149	15.50	96.13	14.9	3.40	4.38	0.02	20.43	0.81	25.23
	98	-24.0	0.41	11.29	27.54	0.125	14.74	84.80	14.0	3.13	4.47	0.09	23.18	0.93	24.92
	99	-24.1	0.35	8.26	23.60	0.102	16.50	61.82	12.2	3.17	3.85	0.10	19.21	0.72	26.68
	100	-24.8	0.73	55.05	75.41	0.095	10.86	87.48	9.7	3.20	3.02	0.18	25.74	0.95	27.09
	101	-24.1	0.72	9.66	13.42	0.125	10.96	114.05	11.1	2.21	5.02	0.01	25.56	1.02	25.06
	102	-24.9	0.65	21.01	32.32	0.177	16.17	109.46	17.6	4.65	3.78	0.50	28.26	1.07	26.41
	103	-25.3	0.57	24.28	42.60	0.126	12.76	98.75	11.6	2.51	4.62	0.08	26.37	1.01	26.11
	104	-24.3	0.38	19.72	51.89	0.131	13.19	99.32	12.6	2.98	4.23	0.14	20.52	0.74	27.76
	105	-26.2	0.35	6.63	18.94	0.150	13.90	107.91	13.6	3.48	3.91	0.82	21.32	0.87	24.51
	106	-24.1	0.38	5.52	14.53	0.135	12.74	105.97	11.5	2.53	4.55	0.00	19.51	0.71	27.48
	107	-24.7	0.34	8.47	24.91	0.124	12.41	99.92	11.4	3.27	3.49	0.10	18.35	0.73	25.14

Table S6. Results of organic carbon isotope composition ($\delta^{13}C_{org}$, ‰), total organic carbon (TOC) contents (wt. %), Hg concentrations (ppb), Hg/TOC ratios (ppb/%), phosphorus (P), aluminum (Al), thorium (Th) and uranium (U) contents (%), P/Al (10^{-4}) and Th/U rations, total sulfur (TS) contents (%), organic C and N element contents (%) and C/N ratios from the studied borehole in the Jiyuan basin of the NCP.

Supplementary References

1. M. Wiedenbeck, *et al.*, Further Characterisation of the 91500 Zircon Crystal. *Geostand. Geoanalytical Res.* **28**, 9–39 (2004).
2. M. Wiedenbeck, *et al.*, Three natural zircon standards for U-Th-Pb, Lu-Hf, trace element and REE analyses. *Geostand. Newsl.* **19**, 1–23 (1995).
3. J. Sláma, *et al.*, Plešovice zircon — A new natural reference material for U–Pb and Hf isotopic microanalysis. *Chem. Geol.* **249**, 1–35 (2008).
4. Y. Liu, *et al.*, In situ analysis of major and trace elements of anhydrous minerals by LA-ICP-MS without applying an internal standard. *Chem. Geol.* **257**, 34–43 (2008).
5. J. Lu, Y. Wang, M. Yang, L. Shao, J. Hilton, Records of volcanism and organic carbon isotopic composition ($\delta^{13}C_{org}$) linked to changes in atmospheric pCO_2 and climate during the Pennsylvanian icehouse interval. *Chem. Geol.* **570**, 120168 (2021).
6. S. Mueller, L. Krystyn, W. M. Kürschner, Climate variability during the Carnian Pluvial Phase — A quantitative palynological study of the Carnian sedimentary succession at Lunz am See, Northern Calcareous Alps, Austria. *Palaeogeogr. Palaeoclimatol. Palaeoecol.* **441**, 198–211 (2016).
7. S. Mueller, M. W. Hounslow, W. M. Kürschner, Integrated stratigraphy and palaeoclimate history of the Carnian Pluvial event in the Boreal realm; new data from the upper triassic kapp toscana group in central Spitsbergen (Norway). *J. Geol. Soc. London.* **173**, 186–202 (2016).
8. J. H. Dembicki, "Source Rock Evaluation" in *Practical Petroleum Geochemistry for Exploration and Production,* (Elsevier, 2017), pp. 61–133.
9. P. A. Meyers, Preservation of elemental and isotopic source identification of sedimentary organic matter. *Chem. Geol.* **114**, 289–302 (1994).
10. P. A. Meyers, Organic geochemical proxies of paleoceanographic, paleolimnologic, and paleoclimatic processes. *Org. Geochem.* **27**, 213–250 (1997).
11. P. A. Meyers, E. Lallier-Vergès, Lacustrine sedimentary organic matter records of Late Quaternary paleoclimates. *J. Paleolimnol.* **21**, 345–372 (1999).
12. J. Fan, *et al.*, Holocene environment variations recorded by stable carbon and nitrogen isotopes of sedimentary organic matter from Dali lake in inner Mongolia. *Quarternary Sci.* **35**, 865–870 (2015).
13. Z. Liu, L. Li, Y. Wang, Late Triassic spore-pollen assemblage from Xuanhan of Sichuan,China. *Acta Micropala eontolofica Sin.* **32**, 43–62 (2015).
14. L. Ji, F. Meng, Palynology of Yanchang Formation of Middle and Late Triassic in Eastern Gansu Province and Its Paleoclimatic Significance. *J. China Univ. Geosci.* **17**, 209–220 (2006).
15. A. M. Zavattieri, N. Mego, Palynological record of the Paso Flores Formation (Late Triassic) on the southeastern side of the Limay River, Patagonia, Argentina. *Ameghiniana* **45**, 483–502 (2008).
16. G. Roghi, P. Gianolla, L. Minarelli, C. Pilati, N. Preto, Palynological correlation of Carnian humid pulses throughout western Tethys. *Palaeogeogr. Palaeoclimatol. Palaeoecol.* **290**, 89–106 (2010).
17. S. N. Césari, C. E. Colombi, A new Late Triasssic phytogeographical scenario in westernmost Gondwana. *Nat. Commun.* **4**, 1889 (2013).
18. S. N. Césari, C. Colombi, Palynology of the Late Triassic Ischigualasto Formation, Argentina: Paleoecological and paleogeographic implications. *Palaeogeogr. Palaeoclimatol. Palaeoecol.* **449**, 365–384 (2016).
19. Z. Fu, X. Yuan, Late Triassic sporopollen assemblage from Liupanshan Basin of Ningxia and their stratigraphical significance. *Acta Palaeontol. Sin.* **37**, 446–454 (1998).
20. T. J. Bralower, H. R. Thierstein, Low productivity and slow deep-water circulation in mid-Cretaceous oceans. *Geology* **12**, 614–618 (1984).
21. S. Liu, S. Su, G. Zhang, Early Mesozoic basin development in North China: Indications of cratonic deformation. *J. Asian Earth Sci.* **62**, 221–236 (2013).
22. J. H. A. Van Konijnenburg-Van Cittert, Osmundaceous spores in situ from the Jurassic of Yorkshire, England. *Rev. Palaeobot. Palynol.* **26**, 125–141 (1978).
23. J. H. A. Van Konijnenburg-Van Cittert, Ecology of some Late Triassic to Early Cretaceous ferns in Eurasia. *Rev. Palaeobot. Palynol.* **119**, 113–124 (2002).

24. L. Li, Y. Wang, Z. Liu, N. Zhou, Y. Wang, Late Triassic palaeoclimate and palaeoecosystem variations inferred by palynological record in the northeastern Sichuan Basin, China. *Palaontologische Zeitschrift* **90**, 327–348 (2016).
25. L. Li, Y. Wang, V. Vajda, Z. Liu, Late Triassic ecosystem variations inferred by palynological records from Hechuan, southern Sichuan Basin, China. *Geol. Mag.* **155**, 1793–1810 (2018).
26. L. Li, Y. Wang, W. M. Kürschner, M. Ruhl, V. Vajda, Palaeovegetation and palaeoclimate changes across the Triassic–Jurassic transition in the Sichuan Basin, China. *Palaeogeogr. Palaeoclimatol. Palaeoecol.* **556**, 109891 (2020).
27. N. Lu, *et al.*, Sedimentological and paleoecological aspects of the Norian–Rhaetian transition (Late Triassic) in the Xuanhan area of the Sichuan Basin, Southwest China. *Palaeoworld* **28**, 334–345 (2019).
28. A. Fijałkowska-Mader, K. Jewuła, E. Bodor, Record of the Carnian Pluvial Episode in the Polish microflora. *Palaeoworld* **00**, 1–20 (2020).
29. R. J. Litwin, Fertile organs and in situ spores of ferns from the late Triassic Chinle Formation of Arizona and New Mexico, with discussion of the associated dispersed spores. *Rev. Palaeobot. Palynol.* **44**, 101–146 (1985).
30. Y. Wang, Fertile organs and in situ spores of Marattia asiatica (Kawasaki) Harris (Marattiales) from the Lower Jurassic Hsiangchi Formation in Hubei, China. *Rev. Palaeobot. Palynol.* **107**, 125–144 (1999).
31. Y. Wang, S. Mei, Fertile organs andin situ spores of a matoniaceous fern from the Lower Jurassic of West Hubei. *Chinese Sci. Bull.* **44**, 1333–1337 (1999).
32. Y. Wang, Fern ecological implications from the Lower Jurassic in Western Hubei, China. *Rev. Palaeobot. Palynol.* **119**, 125–141 (2002).
33. S. Deng, Ecology of the Early Cretaceous ferns of Northeast China. *Rev. Palaeobot. Palynol.* **119**, 93–112 (2002).
34. O. A. Abbink, J. H. A. Van Konijnenburg-Van Cittert, C. J. Van der Zwan, H. Visscher, A sporomorph ecogroup model for the Northwest European Jurassic - Lower Cretaceous II : Application to an exploration well from the Dutch North Sea. *Netherlands J. Geosci. - Geol. en Mijnb.* **83**, 81–91 (2004).
35. Y. Wang, V. Mosbrugger, H. Zhang, Early to Middle Jurassic vegetation and climatic events in the Qaidam Basin, Northwest China. *Palaeogeogr. Palaeoclimatol. Palaeoecol.* **224**, 200–216 (2005).
36. Y. Wang, *et al.*, Fertile structures with in situ spores of a dipterid fern from the Triassic in southern China. *J. Plant Res.* **128**, 445–457 (2015).
